# Extracellular Vesicles in Skin Wound Healing

**DOI:** 10.3390/ph14080811

**Published:** 2021-08-18

**Authors:** Deimantė Narauskaitė, Gabrielė Vydmantaitė, Justina Rusteikaitė, Revathi Sampath, Akvilė Rudaitytė, Gabija Stašytė, María Isabel Aparicio Calvente, Aistė Jekabsone

**Affiliations:** 1 Laboratory of Pharmaceutical Sciences, Institute of Pharmaceutical Technologies, Faculty of Pharmacy, Medical Academy, Lithuanian University of Health Sciences, LT-50162 Kaunas, Lithuania; deimante.narauskaite@lsmu.lt (D.N.); gabriele.vydmantaite@lsmu.lt (G.V.); akvile.rd@gmail.com (A.R.); gabija.stasyte@lsmu.lt (G.S.); maparicio@alumni.usj.es (M.I.A.C.); 2 Preclinical Research Laboratory for Medicinal Products, Institute of Cardiology, Lithuanian University of Health Sciences, LT-50162 Kaunas, Lithuania; justina.rusteikaite@lsmu.lt (J.R.); 20030415@studenti.uniupo.it (R.S.)

**Keywords:** extracellular vesicles, EVs, exosomes, mesenchymal stem cell EVs, plant-derived EVs, wound healing, scaffolds

## Abstract

Each year, millions of individuals suffer from a non-healing wound, abnormal scarring, or injuries accompanied by an infection. For these cases, scientists are searching for new therapeutic interventions, from which one of the most promising is the use of extracellular vesicles (EVs). Naturally, EV-based signaling takes part in all four wound healing phases: hemostasis, inflammation, proliferation, and remodeling. Such an extensive involvement of EVs suggests exploiting their action to modulate the impaired healing phase. Furthermore, next to their natural wound healing capacity, EVs can be engineered for better defined pharmaceutical purposes, such as carrying specific cargo or targeting specific destinations by labelling them with certain surface proteins. This review aims to promote scientific awareness in basic and translational research of EVs by summarizing the current knowledge about their natural role in each stage of skin repair and the most recent findings in application areas, such as wound healing, skin regeneration, and treatment of dermal diseases, including the stem cell-derived, plant-derived, and engineered EVs.

## 1. Introduction

Even though wound healing is a natural self-controlled process, some patients develop a chronic non-healing wound or abnormal scarring [1,2]. As a result, annually, millions of individuals suffer from injuries that are often accompanied by infection. In addition, the ageing population contributes to the increased number of impaired wound healing sufferers, and therefore, a demand for new treatment strategies is growing [3,4]. 

One of the most promising emerging wound healing approaches involves applying extracellular vesicles (EVs) [5]. They are secreted by various cells and appear essential players in regeneration-promoting intercellular communication [6]. EV-based signaling plays a crucial role in all four wound healing phases: hemostasis, inflammation, proliferation, and remodeling [7], suggesting their natural capacity could be exploited to stimulate the impaired healing process. In addition, EVs can be engineered for carrying specific cargo and used for targeted delivery by labelling them with particular surface proteins [8,9,10].

An increasing amount of experimental evidence defines the role of EVs in maintaining skin function, reverting ageing, or disease-related degeneration and immunity disorders, such as atopic dermatitis, hair regrowth, and impaired wound healing [11,12,13,14]. Also, some researchers investigate the regenerative capacity of EVs from sources other than human cell sources, such as milk, plants, or even bacteria. This review aims to promote EV research and regenerative applications by summarizing

(i). the current knowledge about EV involvement in each stage of natural skin repair;
- (ii). the current efforts applying EVs for skin regeneration, wound healing, and treatment of dermal diseases, including the cases of engineered EVs and those of non-human origin.

## 2. The Role of Extracellular Vesicles in Natural Wound Repair

### 2.1. Physiology of Healthy and Wounded Skin 

**Figure 1:**
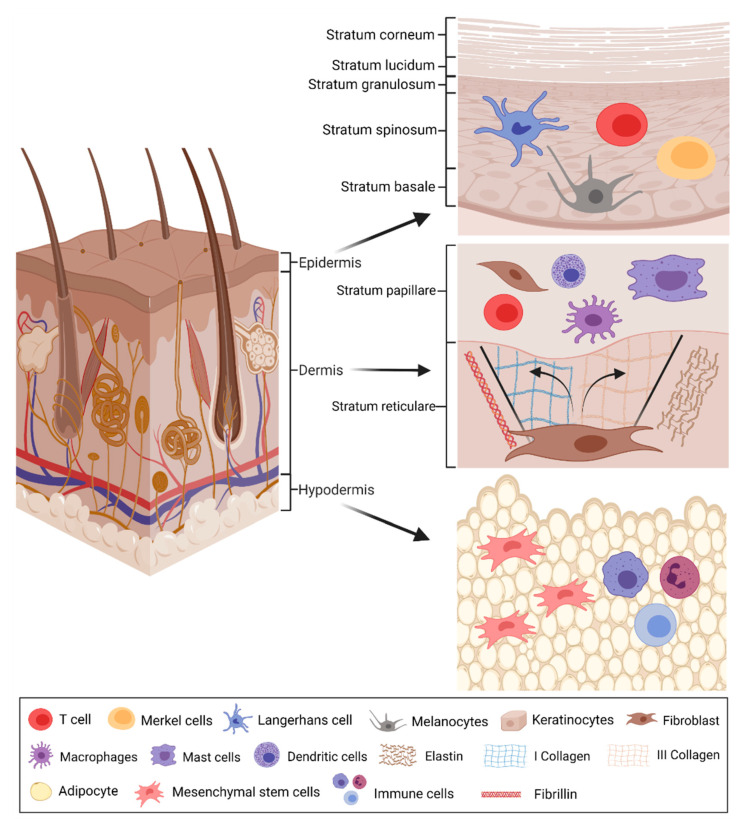
Skin structure. The skin is composed of three layers. The upper layer of the epidermis consists of five micro-layers that are formed by the differentiation of keratinocytes. Melanocytes, Merkel cells, Langerhans cells, and T cells are also present in this layer. The dermis consists of two connective tissue layers that contain sweat glands, hair, hair follicles, muscles, sensory neurons, and blood vessels. The dermal fibroblasts produce an extracellular matrix making the layer rich in elastin, fibrillin, and collagens (I, III). It also has dendritic cells, macrophages, mast cells, and T cells. The lower layer of the hypodermis is rich in adipose cells, mesenchymal stem cells, and immune cells.

Human skin is the largest organ, covering the entire outer surface of the body. It makes a primary barrier against pathogens, UV rays, chemicals, and mechanical damage and regulates body temperature and the amount of water released into the environment. Skin functions are fulfilled by specialized cells found in three anatomically and functionally different skin layers: epidermis, dermis, and hypodermis (Figure 1) [15]. The epidermis consists of 95% keratinocyte cells (KCs) and 5% pigment melanin producing melanocytes. In addition, tactile epithelial Merkel cells, tissue-resident macrophages (Langerhans cells), and T resident memory cells are also present in this layer [16]. KCs are responsible for the formation of the epidermal water barrier by secreting lipids. Moreover, they express Toll-like receptors and produce inflammatory cytokines and chemokines (such as IL-1β, IL-8, and CCL20), which are crucial for pathogen pattern recognition and recruitment of leukocytes under skin injury conditions [17,18,19]. 

The dermis is the next layer after the epidermis and consists of two layers of connective tissue that merge without a clear boundary—stratum papillare and stratum reticulare. The papillary layer is the upper, thinner layer, composed of loose connective tissue, and contacts the epidermis. Next is the retinal layer, which is thicker, less cellular, and composed of dense connective tissue or bundles of collagen fibers [16,20]. The dermis contains sweat glands, hair, hair follicles, muscles, sensory neurons, and blood vessels. The vascular network supports the epidermis, hair follicles, and sweat glands with nutrients. It also plays a central role in the dermal inflammatory response after wounding via the recruitment of neutrophils, lymphocytes, and other inflammatory cells [21]. The dermis also has a groups other than blood-derived immune response cells, such as dermal dendritic cells, macrophages, CD4+ and CD8+ T resident memory cells, mast cells and Foxp3+ T regulatory cells, often located near the hair follicle [16]. The most widely distributed cells in the dermis are fibroblasts primarily responsible for extracellular matrix (ECM) production [22]. They make the layer rich in elastin, fibrillin, collagens (types I and III), and other ECM components, including growth factors. The ECM is responsible for skin mechanical properties such as elasticity and tensile strength. Collagen IV and integrin α6 are critical basement membrane components, contributing to the skin’s physical integrity [16].

The hypodermis, also called the subcutaneous tissue, is the deepest layer of skin and is composed of adipose cells, mesenchymal stem cells (MSCs), blood and lymph vessels [23]. It is endocrinologically and immunologically active. The subcutaneous layer of the skin is predominantly made of adipocytes and mesenchymal fibroblast precursor cells known as pre-adipocytes. The second most common cell type are immune cells, and nearly all immune cell types are included [24]. It contains fat lobes and some skin additives such as hair follicles, sensory neurons, and blood vessels. The subcutaneous adipose tissue underlies and functions as an energy reserve and also a constant source of growth factors to the dermis [25,26]. The adipose tissue MSCs (AdMSCs) are important regulators of tissue homeostasis; they are also explored for scientific and potential clinical purposes [23,27,28,29]. Numerous studies demonstrate that adipose tissue provides a worthy impact on the microenvironment by the secretion of bioactive factors with various functions [23]. The tissue is responsible for energy balance, lipid metabolism, insulin sensitivity, angiogenesis, immunomodulation, and inflammatory response [30].

The synchronized and efficient function of cells from all three layers of skin is needed to restore barrier function after damage. This process is characterized by four classic stages—hemostasis, inflammation, proliferation, and remodeling—which occur one after another, but also overlap. It consists of different cellular and molecular events that require the cooperation of various cell populations [31].

The first response to a wound is a constriction of blood vessels and fibrin clot formation. The most significant cells involved in this process are platelets. These megakaryocyte-derived, non-nucleated cell fragments circulate close to endothelial cells (ECs) during homeostasis. In case of vascular injury, they become activated by agonists released from damaged cells, e.g., collagen, adenosine diphosphate (ADP), von Willebrand factor, fibronectin, thrombin. This activation causes inside-out signaling pathways leading to the actin cytoskeleton and aggregation changes, resulting in platelet plug formation. Simultaneously activated platelets activate coagulation cascades, triggering prothrombin conversion to thrombin, cleaving fibrinogen into insoluble fibrin. Platelet plug and fibrin form a thrombus, which stops the bleeding [31,32,33]. Successful formation of a clot provides a temporary wound matrix for upcoming infiltration of immune cells serving the next phase of wound healing—inflammation. 

Damaged cells and platelets release various “find me” signals (ATP, peptides, ECM components, cytokines, chemokines) at the site of the skin injury. Also, it is often colonized by microorganisms (bacteria and fungi) [34,35], so their molecular motifs are also present to activate immune responses. In the first few hours, the predominant cell line clearing the bed of the wound from remains, damaged cells, and bacteria are neutrophils. They constitute 50% of all cells at the wound site during the first day after tissue injury [36]. Within 48–96 h, monocytes are recruited and evolve into macrophages, whose main functions are phagocytosis of residual tissue and the rise of cytokine secretion and growth factors enhancing inflammatory response [31,37].

During the proliferation phase, granulation tissue is formed by activated fibroblasts, synthesizing ECM components, mainly collagen I. Thus, it provides a platform for new vessels and inflammatory cells [31,38]. A hypoxic state of the wound bed induces vascular EC activation, and this, in turn, initiates angiogenesis. Additionally, endothelial progenitor cells (EPC) are activated and participate in *de novo* blood vessel formation (vasculogenesis) by incorporating into vessels and differentiating into ECs. They are recruited by chemokines and transit to the circulation from the bone marrow, where they reside until vessel injury [39].

Concurrently with the proliferation state, ECM undergoes structural changes while fibroblasts differentiate to contractile myofibroblasts. These processes constitute the last phase of wound healing—remodeling. Ultimately, granulation tissue is replaced by connective tissue and wound healing results in closure [31,37,38].

For this successful restoration of the barrier, physiological functions must occur consistently, with adequate intensity and a specific duration. Any disruptions can cause an impaired wound healing. 

Wounds exhibiting impaired healing usually have disorganized, delayed functioning of participant cells. Such a dysfunction causes pathological pro-inflammatory conditions and chronic wounds. Most cases of such chronic wounds are ulcers caused by diabetes, ischemia, venous stasis disease, or pressure [40].

Multiple local and systemic factors can cause impaired wound healing by affecting one or more phases of the process. Many of these factors are related. Local factors (e.g., oxygenation [41], infections [42]) directly influence the characteristics of the wound itself, while systemic factors (e.g., age [43], hormones [44], stress [45], diabetes [46]) are the overall health or disease state of the individual that affect the ability to heal. For example, the impaired healing in individuals with diabetes involves hypoxia and dysfunction in dermal and epidermal cells. Such healing abnormalities are caused by impaired angiogenesis, high levels of metalloproteases, damage from glycation end-products, and an increase in active oxygen species. In addition, the healing capacity in such cases is further reduced by host immune resistance and neuropathy [40]. Abnormal wound repair may result in various abnormalities, from excessive fibrosis and scarring to underhealing wounds, clinically typified by nonhealing chronic ulcers, posing a significant healthcare challenge [31].

### 2.2. Extracellular Vesicles—Biogenesis, Composition, and Function

Pan and Johnstone were among the first to investigate EVs in 1983, stating that they evolved as a heterogenous family of membrane-surrounded vesicles originating from endosomes or plasma membranes [47]. Data collected over more than 30 years show EV presence almost in all bodily fluids [48,49,50,51,52,53,54] and suggest that EVs mediate cellular metabolism by transferring nucleic acids, lipids, and proteins, acting as signaling mediators in homeostatic or pathological processes [55,56,57].

**Figure 2:**
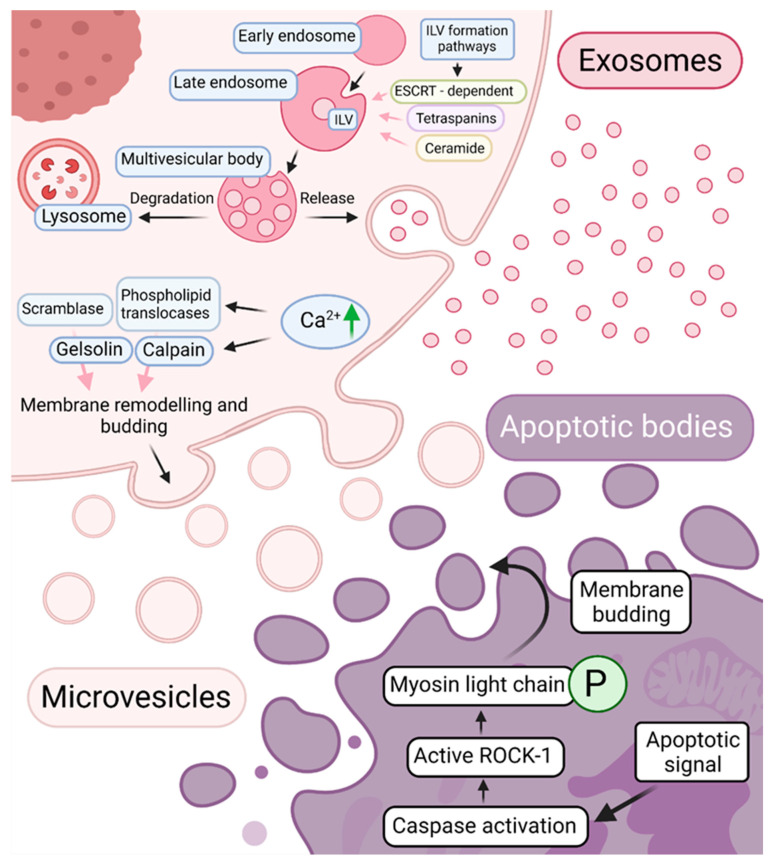
Biogenesis of extracellular vesicles. Upon apoptosis, caspases promote activation of Rho-associated protein kinase 1 (ROCK-1), which phosphorylates myosin regulatory light chain and stimulates actomyosin contractile activity, causing plasma membrane shedding and formation of apoptotic bodies. Exosomes are formed during endosomal sorting. During maturation of an early endosome, intraluminal vesicles (ILVs) are created in ESCRT—dependent or—independent (in the presence of tetraspanins or ceramides) manner. Late endosome with a multitude of ILVs is called the multivesicular body (MVB), which can either diffuse with lysosome for degradation or merge with plasma membrane releasing exosomes. A unique mechanism for microvesicle biogenesis involves Ca^2+^—dependent enzymes—calpain, gelsolin, phospholipid translocases, and scramblase, which promote the distribution of phosphatidylserine (PS) on the outer cell surface resulting in membrane remodeling and subsequent budding.

The term EVs is applied for all secreted membrane vesicles, yet they are highly heterogeneous. According to the available EVs biogenesis data, they can be divided into three subpopulations: apoptotic bodies (50–5000 nm) produced throughout the cell death, exosomes (30–150 nm), formed during endosomal sorting and microvesicles (100–1000 nm in diameter), directly budding from the plasma membrane [58,59,60] (Figure 2).

Upon apoptosis, the cell passes through several morphological events. It starts with chromatin condensation, hereupon membrane budding, which results in the formation of apoptotic bodies with a cytosolic content [61]. Biogenesis of these EVs relies on caspase-mediated activation of Rho-associated protein kinase 1 (ROCK-1). It phosphorylates the myosin regulatory light chain and stimulates actomyosin contractile activity causing plasma membrane shedding of the cytoskeletal network and the formation of an apoptotic body [62,63]. It contains specific membrane rearrangements (e.g., phosphatidylserine (PS) exposure), which, under normal conditions, are recognized by macrophages, and therefore they are eliminated [64].

Newly discovered microvesicles and exosomes currently attract primary research interest. The most complex is the biogenesis of exosomes involving endosomal maturation and sorting machinery. It begins when an early endosome is formed from plasma membrane invagination. Afterwards, the endosome travels to the center of the cell, gradually changing the composition of the load it carries and the rearrangement of the membrane [65,66]. During this process, the early endosome transforms into the late endosome aggregating so called ”intraluminal vesicles” (ILVs). They are formed in the presence of multisubunit machinery—endosomal sorting complex required for transport (ESCRT)—which carries out budding and scission of the endosomal membrane. This canonical ESCRT pathway can intersect with the generation of ILVs carried out by other proteins. For example, protein syntenin combined with ESCRT accessory protein ALIX (ALG-2-interacting protein X) can engage cargos with the ESCRT-III complex proteins and promote membrane bending [67]. Besides, ESCRT-independent mechanisms also exist. They include the participation of membrane proteins tetraspanins [68,69] and sphingolipid ceramide [70,71]. In the process of ILV formation, cytosolic proteins, nucleic acids, and lipids are recruited. As the number of ILVs increases, the late endosome matures into the multivesicular body (MVB). Once formed, it either fuses with lysosome for degradation or with the cellular membrane releasing the ILVs as exosomes into the extracellular space [72].

The mechanisms of microvesicle biogenesis are still not understood. Some molecular mechanisms involved in the stages of EV biogenesis are common to both exosomes and microvesicle formation. These include the action of ceramide formed by sphingomyelinase and ESCRT proteins [73]. However, the component of ESCRT-I complex—tumour susceptibility gene protein 101 (TSG101)—can also participate in mechanistically different membrane budding from ILV formation. It was shown that TSG101 could be recruited to the cell surface by arrestin domain-containing protein 1 and promote direct membrane invagination [74]. Moreover, a unique mechanism of microvesicle biogenesis can be membrane phospholipid asymmetry rearrangement. It is mediated by Ca^2+^-dependent enzymes—calpain, gelsolin, phospholipid translocases, and scramblase, which promote the distribution of PS on the outer cell surface. Such membrane remodeling results in physical membrane flexion and actin skeletal restructuring leading to microvesicle detachment [75].

The protein composition of EVs in most cases depends on the mode of biogenesis. For instance, exosomes tend to be more enriched in tetraspanins CD37, CD53, CD63, CD81, CD82 [76,77], and ESCRT-associated proteins, such as TSG101, ALIX, and syntenin [67,78]. Moreover, chaperones, such as heat shock cognate 71 kDa and heat shock protein 90 (Hsp90), are abundantly found in exosomes. Data suggest that these proteins might promote the incorporation of cytosolic components to the exosomal membrane [79]. Additionally, 14-3-3 epsilon and pyruvate kinase M2 found the exosomes of most cell types, also contribute to protein sorting into exosomes [80]. Due to their plasma membrane origin, microvesicles tend to be enriched in proteins of a different repertoire, including integrins, P-selectin, and glycoprotein Ib [76,81]. Moreover, they carry more proteins with posttranslational modifications, such as glycoproteins or phosphoproteins, compared to exosomes [82]. Lastly, apoptotic bodies contain DNA-binding histones and are depleted in glycoproteins, which is in direct contrast to exosomes [83,84].

Irrespective of cell origin, proteins like tetraspanins, ALIX, TSG101, and heat-shock chaperones are commonly found in all EV subpopulations. They can consequently be used as general EVs markers [77,83]. In contrast, proteins within the mitochondria (e.g., aconitase), Golgi apparatus (e.g., GM130), endoplasmic reticulum (e.g., calreticulin), and some cytoplasmic proteins (e.g., α-tubulin) have appeared to be depleted in EVs isolated by differential centrifugation [85,86]. Overall, the absence might provide additional confirmation of the purity of the EV preparation.

The protein composition of different EV subtypes shows a substantial overlap, even if some proteins are more common in one than in another EV subtype [82]. In addition to this, overlapping sizes and similar morphology makes it challenging to characterize different EV types fully and standardize isolation methods. For these reasons, it is often unclear which subpopulation is responsible for any particular effect and articles exploring EV role focus on the potential functions rather than their origins. Therefore, specific markers for different EVs subpopulations and new standardized isolation methods remain to be determined.

Among other proteins, EVs might contain enzymes involved in lipid metabolism. For example, under hypoxic conditions, adipocytes secrete EVs containing increased levels of enzymes that participate in *de novo* lipogenesis, such as fatty acid synthase. After treatment with such EVs, adipocytes consume more lipid molecules, primarily because of fatty acid synthase delivered to cells by the EVs [87]. Additionally, EVs from RBL-2H3 basophil cells transport GTP dependent phospholipases D and A2 and bioactive lipids such as arachidonic acid and prostaglandin E2 [88].

Furthermore, EVs can transport nucleic acids: DNA within the size of 100 base pairs to 2.5 kilobase pairs [89,90,91,92] and RNA of fewer than 200 nucleotides. [50,93]. The DNA cargo in the EVs reflects the parental cells’ genomic DNA. For example, common gene mutations in cancer cells such as mutations in BRAF, epithelial growth factor receptor, KRAS and p53 were successfully detected in EVs derived from melanoma and pancreatic cancer cells [92]. Moreover, RNA was functional after EV-mediated transfer. Polyadenylated mRNAs participated in translation after uptake, which has been proved in recipient cells by translation assays [94,95,96]. The miRNAs transported between cells by EVs may regulate the translation of target mRNAs in recipient cells [94,97]. For example, EVs released from T cells can transfer specific miRNAs (such as miR-335) to recipient antigen-presenting cells [94].

Interestingly, some RNAs are systematically present in EVs, suggesting a specific mechanism of RNA incorporation into the vesicles [57,95,96]. Most likely, RNA is sorted into EVs due to particular sequence motifs. Indeed, the sequence motif of CUGCC in the binding site for miR-1289 was shown to promote the miRNA recruitment into EVs [98]. Studies suggest that this process might be carried out by heterogeneous nuclear ribonucleoprotein A2B1 (hnRNPA2B1), recognizing these specific sequence motifs and promoting miRNA packaging. In addition, specific miRNA binding is supervised by posttranslational modification of hnRNPA2B1 in the form of SUMOylation, which regulates protein stability and cellular trafficking [99]. The classification of miRNAs into EVs can also be driven by 3′ end posttranscriptional modifications because 3′ adenylated miRNAs are prevalent in cells, whereas 3′ uridylated miRNAs are characteristic of EVs miRNAs [100]. This data shows regulation after protein translation or transcription can modulate specific miRNA packaging into EVs.

Overall, findings have shown the substantial importance of EVs as an intercellular communication mediator.

### 2.3. Physiological Role of Extracellular Vesicles in Wound Healing

Recent in vivo studies reported EV presence in wounds and acknowledged their participation in normal healing [7,40]. This section will summarize the latest findings on EVs derived from “key-player” cells in skin repair under physiological conditions. The main results on the topic are listed in Table A1.

#### 2.3.1. Extracellular Vesicles in Hemostasis

**Figure 3:**
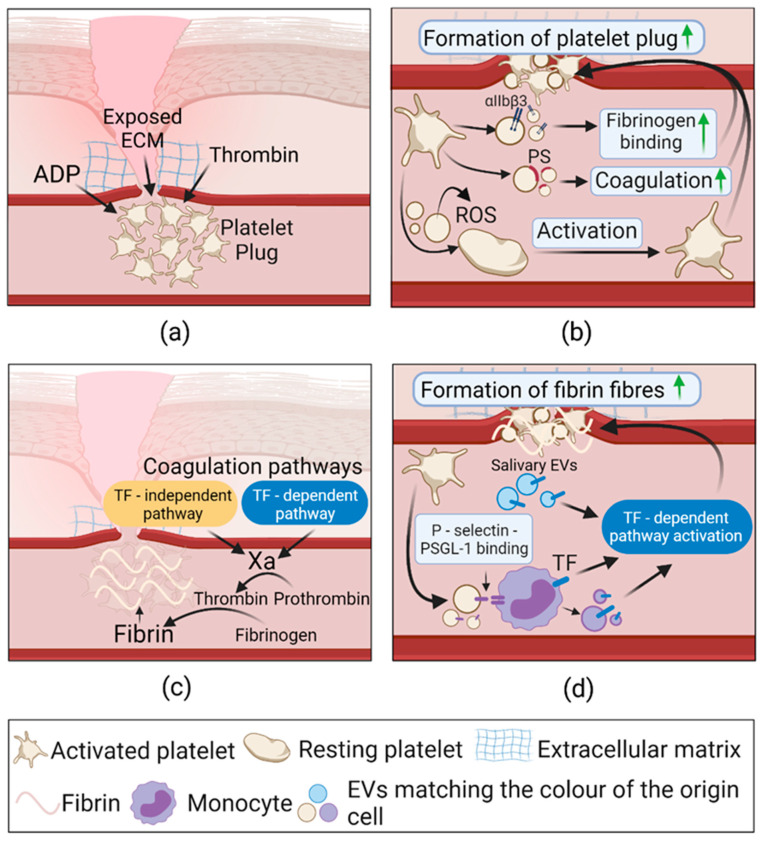
Supposed EV role in hemostasis phase of healthy wound healing. (**a**)—Platelet activation upon skin injury. Damaged cells release danger-signaling molecules such as ADP, collagen, and thrombin, causing changes in platelet cytoskeleton and inducing platelet plug formation, which temporarily stops the bleeding. (**b**)—Release of pro-coagulant EVs (PEVs) from activated platelets. PEVs’ pro-coagulant property is thought to be due to: an active form of αIIbβ3 integrin, having greater affinity to fibrinogen; (ii) exposure of phosphatidylserine (PS), which provides a platform for coagulation factors, (iii) transfer of reactive oxygen species (ROS) producing NOX-1, which enhances platelet activation. (**c**)—Formation of fibrin fibers. Tissue factor (TF) dependent and independent coagulation cascades meet in a common pathway, which results in fibrinogen conversion to fibrin. It binds to aggregated platelets and forms a thrombus. (**d**)—EV role in fibrin formation. TF-dependent coagulation pathway can be induced by PEVs transferring P-selectin, which causes TF exposure on monocyte membrane upon PSGL-1 binding. Alternatively, TF can be introduced by salivary and monocyte-derived EVs. The illustration is a simplified depiction based on the latest findings (see Table A1).

The EV role in hemostasis is schematically depicted in Figure 3. The most abundant EVs in blood circulation are platelet-derived EVs (PEVs) [101], and they significantly contribute to the regulation of hemostasis [60,102]. PEV procoagulant function mostly depends on platelet activation, e.g., PEVs from thrombin-activated platelets were more efficient in clot formation (*p* < 0.01) than PEVs from resting-state platelets (*p* < 0.05). PEV involvement in clot formation is mediated by the activated form of integrin αIIbβ3, which provides high affinity to fibrinogen and results in the construction of fibrin clots [103]. Additionally, activation of P2Y12 platelet receptors by ADP induces the release of PEVs, exposing the proinflammatory P-selectin and procoagulant PS [104]. The latter serves as a platform for procoagulant factors and promotes the generation of thrombin [105]. However, the plasma containing PEVs with exposed PS does not clot without the tissue factor (TF), which is known to be the primary initiator of blood coagulation. Upon vascular injury, TF forms a complex with coagulation factor VIIa, which, in turn, activates factor X, responsible for prothrombin conversion to thrombin [31]. In another study of sepsis-induced platelet activation, PEVs were shown to bind TF and Factor XII and evoke the formation of thrombin only in the presence of Factor VII and XII. The finding indicates that PEVs mediates both extrinsic (TF-dependent) and intrinsic (TF-independent) coagulation pathways [106]. Although TF presence in the PEVs of healthy individuals is still disputed [107,108], they can induce procoagulant effects indirectly by binding P-selectin to P-selectin glycoprotein ligand-1 (PSGL-1) on monocytes and cause TF exposure on their surface [38]. As an alternative, TF can be transferred via monocyte-derived EVs [109]. Interestingly, TF was also found in salivary EVs, implying their ability to facilitate hemostasis at the skin injury site upon the licking reflex [110].

In addition to the above-described coagulation factor transferring, a novel mechanism was proposed, introducing PEV ability to communicate components of NADPH oxidase (NOX-1). This study reported that PEVs from activated platelets generating superoxide in a NOX-1-dependent way, affect other platelets and enhance fibrinogen binding. It was also suggested that PEVs induce platelet activation via collagen receptor—GPVI [111].

The increasing amount of data shows that the EVs of various origins cooperate to ensure the successful formation of a platelet plug and fibrin fibers, which serve as a platform for subsequent infiltration of immune cells.

#### 2.3.2. Extracellular Vesicles in Inflammation

The studies of neutrophil-derived EVs (NDEVs) show that they exert anti- and pro-inflammatory functions depending on environmental factors persisting at the time of EV biogenesis. It was reported that upon infectious conditions, NDEVs increase the production of ROS (*p* = 0.0371) and IL-8 (*p* = 0.0014) in other neutrophils. Additionally, they boost the expression of adhesion molecules E-selectin and VCAM-1 on ECs, indicating their activation. In contrast, resting-state NDEVs do not affect endothelium or alleviate its activation. Interestingly, EVs from apoptotic neutrophils also distinguish themselves with a potent procoagulant effect [112]. Another study has shown that EVs from activated neutrophils can also act in their own respect without transferring cargo to recipient cells. Evidently, NDEVs carry NOX-2 and, after directly interacting with pathogen-associated molecular patterns (PAMPs), increase ROS production in a receptor-dependent fashion *p* < 0.05). However, this does not apply to EVs from endothelium-attached neutrophils, which indicates NDEV heterogeneity due to neutrophil interaction with other cells. In addition, both NDEV subsets (from adherent and non-adherent neutrophils) generate leukotriene B4 (LTB4) and migrate towards a chemotactic gradient. ROS is a known protector against pathogens and can stimulate protective signaling pathways in other cells, while LTB4 is a chemo-attractant. These findings show that activated NDEVs mediate inflammation by producing “danger signals”. Additionally, adherent NDEVs activate pro-inflammatory gene expression in human umbilical vein endothelial cells (HUVECs), whereas non-adherent NDEVs act the opposite, promoting anti-inflammatory gene expression [113].

**Figure 4:**
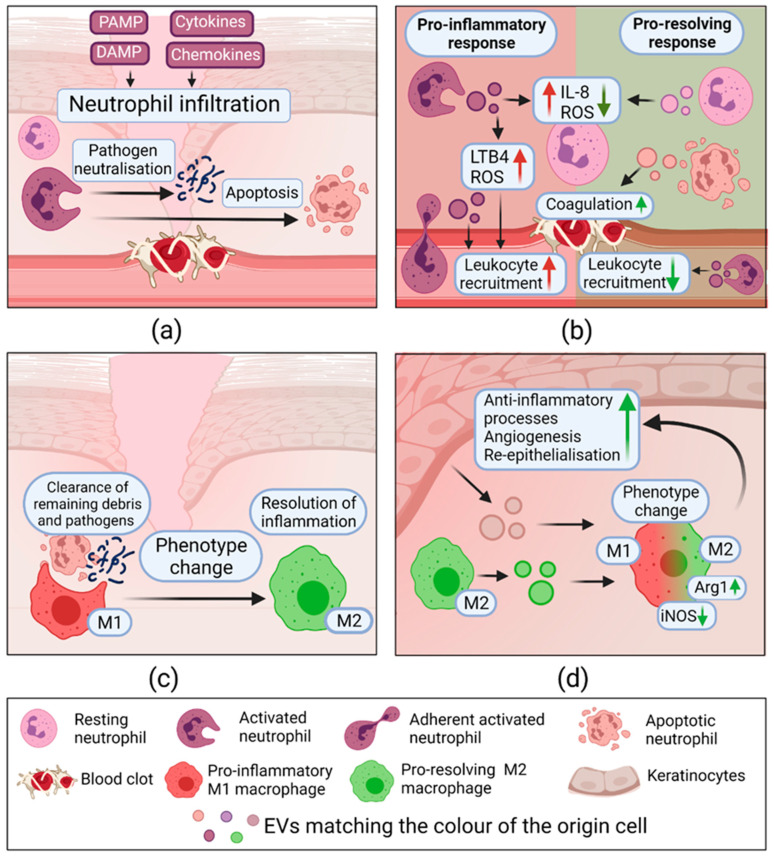
The role of extracellular vesicles (EVs) during the inflammation phase of wound healing. (**a**) Neutrophil cell recruitment. First immune cells to be recruited to the wound site are neutrophils. They respond to signals provided by damaged cells, microbes, and platelets (PAMP—pathogen-associated molecular patterns; DAMP—damage-associated molecular patterns; cytokines and chemokines). After they clear the wound of pathogens and cell remains, they become apoptotic. (**b**) Neutrophil–derived EVs’ (NDEVs) function depends on environmental conditions. Activated-state NDEVs promote reactive oxygen species (ROS), interleukin 8 (IL-8) production in other neutrophils, as well as directly induce ROS and leukotriene B4 synthesis in their turn. This results in the maintenance of a pro-inflammatory environment. In contrast, resting-state NDEVs act the opposite, while apoptotic NDEVs promote coagulation. Additionally, endothelium-attached NDEVs induce pro-inflammatory gene expression, while non-adherent NDEVs induce anti-inflammatory genes in endothelial cells. (**c**) Macrophage cell recruitment. Macrophages infiltrate the wound site and destroy remaining pathogens and apoptotic neutrophils. Pro-inflammatory M1 macrophages shift their phenotype to pro-resolving M2 phenotype. (**d**) EVs’ activity in macrophage phenotype change. Macrophages change phenotype when EVs from pro-resolving macrophages or wound edge keratinocytes transmit their active cargos. Consequently, the levels of inducible nitric oxide synthase (iNOS) and arginase (Arg1), M1 and M2 macrophage markers, are in control. Reprogrammed macrophages accelerate the transition to the proliferative stage of wound healing. The illustration is a simplified depiction based on the latest findings (see Table A1).

During the inflammatory phase of wound healing, macrophages have an essential role in transitioning from the inflammatory phase to the proliferative one. Macrophages undergo a phenotype change: from possessing pro-inflammatory properties to pro-resolving/healing properties, also referred to as M1 and M2 phenotypes, respectively [37,114]. The effect of EVs derived from macrophages of different phenotypes on cutaneous healing has been recently analyzed. It was found that M2 macrophage-derived EVs (M2-EVs) induce macrophage reprogramming from M1 to M2 phenotype; M2-EVs cause a complete absence of M1 marker—inducible nitric oxide synthase (iNOS) but induce M2 marker arginase—expression. Moreover, CCL24, CCL22, and MFG-E8 cytokines are identified as the main EVs compounds responsible for cell reprogramming [115]. Interestingly, EVs from wound edge keratinocytes (KCs-EVs) exhibit a similar role in the phenotypic change of macrophages. A brilliant study by Xiaoju Zhou and colleagues reported that these EVs, unlike uninjured skin EVs, expose a characteristic N-glycan composition on their surface and promote their uptake by wound macrophage cells. Consequently, wound edge KCs-EVs downregulate pro-inflammatory iNOS, CD74, TNF-α genes, and upregulate anti-inflammatory CL3, and this causes reprogramming to pro-resolving macrophage phenotypes. Moreover, the authors demonstrated that knocking down hnRNPA2B1 responsible for miRNA packing to EVs in KCs causes impaired wound closure and persistence of pro-inflammatory iNOS expressing macrophages in vivo. Essentially, miRNA packaging in KCs-EVs is crucial for resolving wound inflammation [7]. Once macrophages are reprogrammed, they accelerate fibroblast migration and ECs tube formation [115]. The inflammation phase and the role of EVs are depicted in Figure 4.

All this data indicates that EVs exert diverse anti- and pro-inflammatory effects modulating the inflammatory response. In addition, the stimulation of macrophage reprogramming has a vital role in the transition to the proliferating phase of wound healing.

#### 2.3.3. Extracellular Vesicles in Proliferation

**Figure 5:**
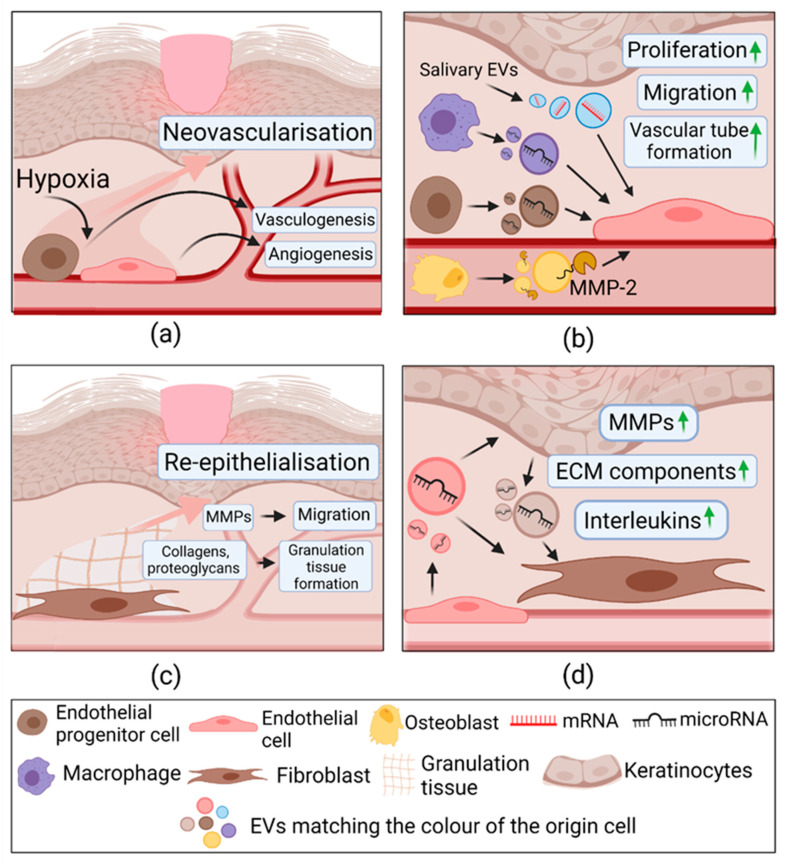
The role of extracellular vesicles (EVs) during the proliferation phase of wound healing. (**a**) Neovascularization. The injury site is in a state of hypoxia, therefore inducing activation of endothelial cells (ECs) and recruitment of endothelial progenitor cells (EPCs), which promote new vessel formation by two mechanisms—angiogenesis and vasculogenesis, respectively. (**b**) The variety of EVs contributes to neovascularization. Synthesis of critical pro-angiogenic factors is promoted by EVs derived from saliva, macrophages, EPCs, and osteoblasts. They stimulate ECs migration, proliferation, and vascular tube formation by transferring different cargos (mRNA, miRNA, MMPs). (**c**) Re-epithelialization. Fibroblasts are “key player” cells in this process. They clear a path by secreting matrix metalloproteinases (MMPs) and migrate towards the wound site, where they synthesize collagen, proteoglycans, and other granulation tissue comprising components. (**d**) EV role in re-epithelialization. EVs mediate crosstalk between ECs, keratinocytes (KCs), and fibroblasts. By transferring miRNA, EC, and KC-derived EVs, this promotes the release of extracellular matrix (ECM) components, MMPs involved in fibroblast migration, and interleukins promoting angiogenesis, KC, and macrophage migration. The illustration is a simplified depiction based on the latest findings (see Table A1).

Under wound healing conditions, the injury site is in hypoxia, therefore inducing activation of local vascular ECs. Once they are activated, the angiogenesis process begins, in which new blood vessels form from the existing ones. Some of the most critical angiogenic signals are Vascular Endothelial Growth Factor (VEGF), Fibroblast Growth Factor (FGF-1), and angiopoietins. In response to them, ECs increase permeability for extravasation of plasma proteins, which deposit provisional ECM. Proteolytic degradation carried out by matrix metalloproteinases (MMPs) remodels this ECM and enables migration of ECs, liberated from the basement membrane [116]. Additionally, EPCs are activated and participate in *de novo* blood vessel formation (vasculogenesis) by incorporating into vessels and differentiating into ECs. They are recruited by chemokines and transit through the circulation from the bone marrow, where they reside until vessel injury [39]. The proliferation phase and the role of EVs are represented in Figure 5.

Recently, studies have shown that EPCs’ released paracrine factors can induce activation of tissue-resident EC and suggest that this mechanism might be more significant in new vessel development than their direct differentiation [117]. Indeed, EVs from umbilical cord-derived EPCs induce pro-angiogenic effects in in vitro and in vivo healthy and diabetic rat wound models. They up-regulated a broad range of pro-angiogenic factor expression in vascular ECs; some of them include E-selectin, angiopoietin, FGF-1, cyclooxygenase 2 (COX-2), and cell cycle activator c-Myc [118,119]. The authors demonstrated that this effect depends on ERK1/2 signaling and speculated that miR-21, found in EVs, might be the culprit of its activation [119]. Moreover, EVs from bone-marrow-derived EPCs are enriched in miRNA-221-3p, which increases the expression of pro-angiogenic factors, including adhesion molecule PECAM-1 (*p* < 0.01), VEGF (*p* < 0.05), and cell proliferation marker Ki67 (*p* < 0.05) [120]. These findings suggest that EPCs-derived EVs (EPCs-EVs) promote angiogenesis by inducing ECs proliferation, motility, and tube formation.

However, EVs of an origin other than endothelial origin can also contribute to angiogenesis. For instance, EVs from macrophages (M-EVs) contain even higher concentrated VEGF, Wnt3a, and miR-130a than their parent cells, and some levels of miR-210 and miR-126 were also identified. These factors are known contributors to EC angiogenic activity and, as the authors suggest, might be responsible for EC proliferation, migration, and tube formation induced by M-EVs [121,122]. Furthermore, an interesting study of mature osteoblast-derived EVs demonstrated angiogenic capacities through the VEGF/ERK1/2 signaling pathway. It was shown that they carry metalloproteinase-2 (MMP-2), which is crucial for angiogenesis as it degrades ECM components that facilitate ECs migration [123]. Bobin and colleagues showed similar effects on angiogenesis of salivary EVs, yet demonstrated a novel mechanism of action. Saliva-EVs transfer mRNA of ubiquitin-conjugated enzyme E2O (UBE2O) that is overexpressed in ECs. UBE2O participates in ubiquitin-mediated proteolysis and decreases levels of SMAD6, a signal transducer known to be an angiogenesis suppressor. This effect resulted in pro-angiogenic cytokine bone morphogenetic protein 2 upregulation [124]. These discoveries revealed that macrophages, bone-forming cells, and saliva upon wound licking promote vessel formation and contribute to the wound healing process [121,122,123,124].

Fibroblasts play a vital role in skin structure formation. They clear a path by secreting proteases and migrate towards the wound site, where they synthesize collagen, proteoglycans, and other granulation tissue comprising components [38]. ECs-EVs can contribute to the process by mediating ECs-fibroblast or ECs-KCs crosstalk. EC-EVs derived from the plasma of healthy volunteers induce migration and proliferation and prevent senescence in diabetic skin fibroblasts through PI3K/Akt/mTOR signaling pathway. Additionally, in fibroblasts and epidermal keratinocyte-like cells (HaCaT), they promote nuclear translocation of transcriptional regulator Yes-associated protein (YAP) and subsequently, activation of its downstream effector—connective tissue growth factor (CTGF). This regulatory axis is known to participate in collagen deposition, fibroblast proliferation, and differentiation to myofibroblasts, which are critical in the remodeling phase of wound healing [125,126].

Notably, KCs-EVs strongly influence fibroblasts and may regulate several features in wound healing [127,128]. Ping Huang and colleagues reported that KC-EVs activate various signaling pathways, with the most prominent effect on ERK1/2. This pathway mediates induction of pro-migratory (MMP-1, MMP-3) and pro-angiogenic/pro-inflammatory (IL-6, IL-8) gene and protein level expression. Moreover, KCs-ECs suppress the expression of the MMP inhibiting proteins RECK and TIMP [128]. More than a third of genes regulated by KC-EVs participate in the signaling of transforming growth factor β (TGF-β), a vital contributor to wound healing. These molecular changes increase fibroblast migration and stimulate them to produce the endothelial tube formation promoting factors [127]. Authors also showed that a critical candidate for fibroblast regulation in KCs-EVs might be miR-21 [128].

These articles suggest that EVs released from cells during physiological wound healing contribute to neovascularization and epidermal layer reconstruction, which overlaps with the last healing phase—remodeling.

#### 2.3.4. Extracellular Vesicles in Remodeling

**Figure 6:**
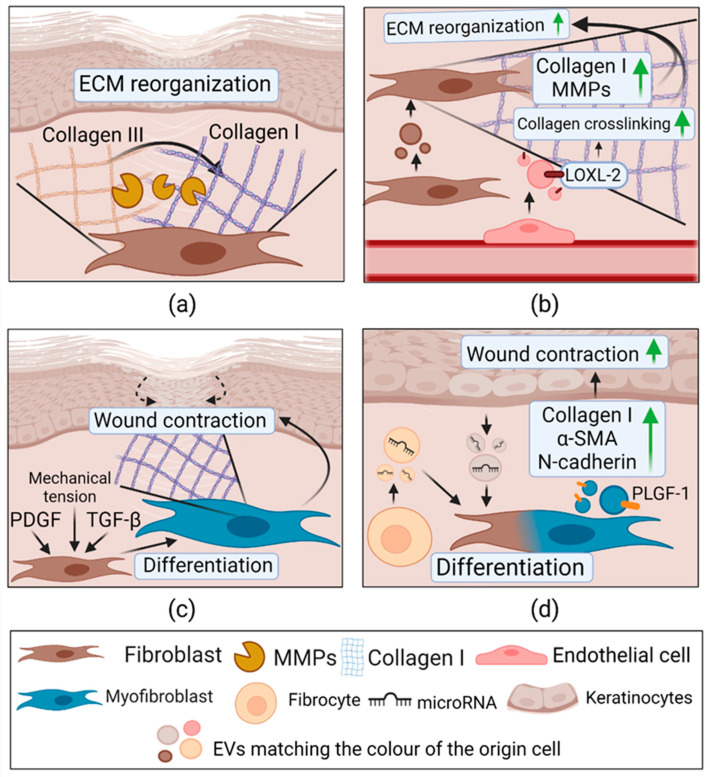
The role of extracellular vesicles’ (EVs) role during the remodeling phase of wound healing. (**a**) Extracellular matrix (ECM) reorganization. Type III collagen, largely expressed in early granulation tissue, is replaced by dominant skin collagen—type I. For its reorganization, collagen and other ECM components are cleaved by matrix metalloproteinases (MMPs). “Key players” in this process are fibroblasts. (**b**) EVs’ role in ECM reorganization. Synthesis and modifications of crucial ECM reorganization components are activated by fibroblast and endothelial cell-derived EVs. Latter ones provide lysyl-oxidase-like 2 (LOXL-2) enzyme, catalyzing collagen crosslinking, and restoring tensile strength. (**c**) Myofibroblasts promote wound closure. PDGF, TGF-β, and mechanical tension initiate fibroblast differentiation to myofibroblasts, synthesizing large amounts of collagen I and promoting wound contraction. (**d**) EVs’ role in fibroblast differentiation. Both keratinocyte and fibrocyte-derived EVs carry miRNA and induce fibroblast differentiation to myofibroblast by increasing collagen I, α-SMA, and N-cadherin expression. In addition, myofibroblasts release EVs, which also contribute to wound closure by carrying placental growth factor 1 (PLGF-1). The illustration is a simplified depiction based on the latest findings (see Table A1).

The last phase of wound healing and EV’s importance in it are illustrated in Figure 6. Type III collagen is mainly synthesized in the early stages of wound healing, but eventually, it is replaced by type I—the dominant fibrillar collagen in the skin. During ECM reorganization, these components are specifically cleaved by MMP-1, MMP-8, and for final collagen maturation, it is modified by lysyl oxidase (LOX), resulting in covalent cross-linking and restoration of tensile strength [129]. Unsurprisingly, fibroblast-derived EVs contribute to ECM reorganization by increasing collagen I, MMP-1, and MMP-3 gene expression (*p* < 0.01) in other fibroblasts. This effect assists in migration and collagen deposition increase (*p* < 0.01) [130]. Furthermore, the study of Olivier G. de Jong and colleagues demonstrated ECs-EVs’ direct effect on ECM remodeling. It was shown that under hypoxic conditions, ECs release EVs exposing LOX family member lysyl oxidase-like 2 (LOXL2), which facilitates collagen I crosslinking and promotes collagen gel contraction [131].

Mechanical tension, TGF-β, and platelet-derived growth factor (PDGF) are considered to be initiators of fibroblast differentiation to a contractile, α smooth muscle actin (α-SMA) expressing myofibroblasts. Importantly, they synthesize large amounts of collagen I [132]. In addition to KCs-EV’s role in the proliferation phase, they also participate in remodeling by initiating the fibroblast differentiation. The treatment with KC-EVs upregulates gene expression and protein level of two known myofibroblast markers—α-SMA and N-cadherin [128]. A recent study showed that EVs from normal skin wound myofibroblasts stimulated collagen I production in cutaneous fibroblasts. This effect was caused by VEGF family member—placental growth factor 1 (PLGF-1)—abundantly found in myofibroblast EVs [133]. Moreover, a study by Adolf Geiger and colleagues showed a significant fibrocyte-derived EV (FDEV) role in wound healing [134]. These progenitor cells originate from bone marrow and acquire myofibroblast-like properties upon injury [135]. Evidence shows that FDEVs carry components such as Hsp-90α, total and activated signal transducer, and activator of transcription-3 (STAT3) [134]. Secreted HSP-90α is characterized by unique properties of promoting cell motility and re-epithelialization. It binds lipoprotein receptor-related protein-1 and activates the Akt signaling pathway [136]. Additionally, STAT3 can activate a broad range of signaling cascades regulating ECM remodeling, angiogenesis, and chemotaxis [137]. Besides these components, FDEVs are enriched in anti-inflammatory (miR124a, miR-125b), pro-angiogenic (miR-126, miR-130a, miR-132), and collagen deposition regulating (miR-21) mi-RNAs. Lastly, FDEVs increase (*p* < 0.01) α-SMA and collagen I expression in fibroblasts, most likely leading to differentiation [134].

The above-described evidence highlights the role of EVs in each wound healing phase. However, in the case of pathological wounding, their application has similar drawbacks. For example, EV assistance in coagulation or inflammation phases depends on specific cells‘ activation or interaction with other cells. Namely, the procoagulant role of PEVs relies on the activation of platelets with different stimulants (ADP, thrombin, collagen). Additionally, TF presence in EVs released from activated platelets remains unclear, meaning that EVs from these cells alone might not necessarily lead to coagulation, as well as complete wound healing. Moreover, pro-/anti-inflammatory functions of NDEVs may depend on neutrophil contact with ECs. In contrast, fibroblasts alone secrete EVs, which promote successful wound healing by activating several crucial processes. By transferring miR-21 and primarily activating ERK1/2 signaling pathways, the EVs induced angiogenesis, ECM reorganization, and differentiation to myofibroblasts, promoting wound contraction. The same miRNA and many others were detected in stem cells derived from bone marrow, specifically EPCs-EVs and FDEVs. Thus, their overall effect on wound healing is undoubted. For this reason, in the next chapter, we summarize the current evidence about the role of EVs, mostly from bone marrow-derived MSCs (BMSCs) and AdMSCs in skin barrier repairing.

## 3. Stem Cell-Derived Extracellular Vesicles in Skin Wound Healing

**Figure 7:**
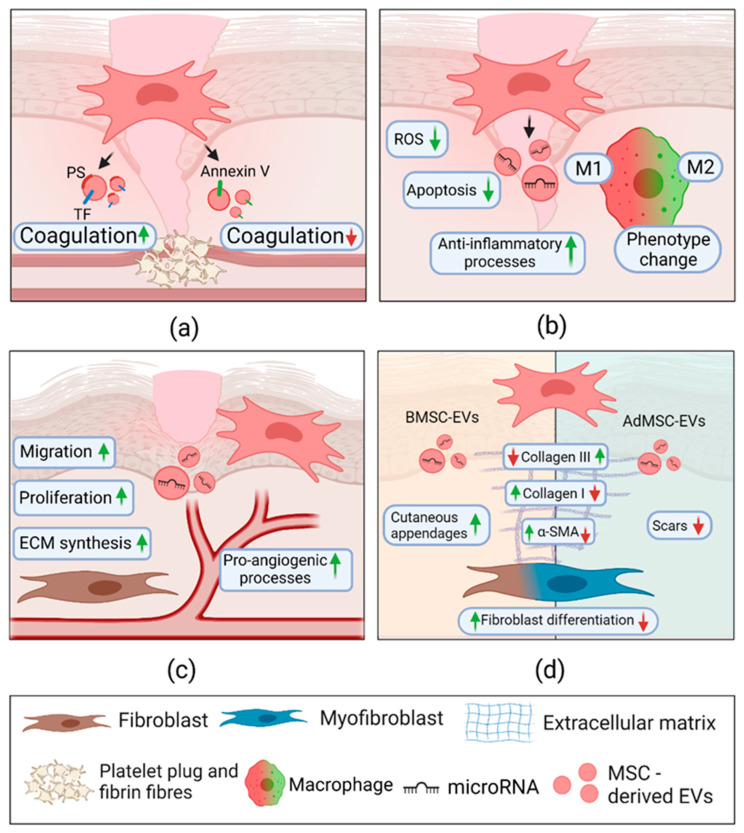
The role of mesenchymal stem cell-derived extracellular vesicles (MSC-EVs) in wound healing. (**a**)—MSC-EVs in hemostasis. MSC-EVs contain pro- and anticoagulant factors, which balance and regulate blood coagulation. (**b**)—MSC-EVs in inflammation. MSC-EVs support anti-inflammatory processes, reducing reactive oxygen species (ROS) synthesis, alleviating apoptosis, and inducing macrophage phenotype change from pro-inflammatory (M1) to anti-inflammatory (M2). (**c**)—MSC-EVs in proliferation. MSC-EVs stimulate fibroblast migration and proliferation to the wound site, resulting in raised levels of extracellular matrix (ECM) components synthesis. Also, MSC-EVs can promote vascularization. (**d**)—MSC-EVs in remodeling. Bone marrow MSC-EVs (BMSC-EVs) increase collagen I production, α-smooth muscle actin (α-SMA) and fibroblast differentiation to myofibroblast; however, they decrease collagen III synthesis. Besides, BMSC-EVs boost new cutaneous appendage formation. Adipose mesenchymal stem cell extracellular vesicles (AdMSC-EVs) act opposite and lead to scar reduction.

MSCs are multipotent mesenchymal stromal cells, which can differentiate into diverse cell types, for instance, adipocytes, osteocytes, chondrocytes, and ECs [138]. Due to immunosuppressive, anti-inflammatory, tissue recovering, and differentiation stimulating properties of the MSCs, they are used for cell therapy in regenerative medicine [139]. Cell therapy is based on injured tissue replacement and restoring of its biological functions [140]. However, using MSCs have some drawbacks: the requirement for a consistent source of stable phenotypic cells, a risk of immunological rejection and risk of tumour development [138]. Nevertheless, recent studies indicate that MSCs modulate tissue regeneration through released paracrine factors, and among them, EVs play a vital role [140]. They participate in main wound healing phases: help prevent inflammation, induce cell proliferation, new tissue formation, and maturation by transferring various biomolecules. Nowadays, MSC-derived EVs are considered novel non-cellular therapy, which can cut the safety limitations of cell therapy [140,141]. The effects of MSC-EVs on hemostasis are summarized in Table A2 and Figure 7.

### 3.1. Mesenchymal Stem Cell-Derived Extracellular Vesicles in Hemostasis

As described above, wound healing starts with blood clot formation, which leads to organism prevention and protection from loss of blood. It is a dynamic process based on platelet aggregation [142]. It is known that MSC-derived EVs have procoagulant properties, usually depending on their transferred cargo. In most cases, MSC-EV cargo resembles that of skin cell EVs released during wound healing.

EVs from MSCs might affect blood coagulation. Silachev with colleagues showed that in the presence of umbilical cord MSC-EVs, human blood clot formation time and lag period of spontaneous clotting is significantly reduced compared to the EV untreated group [143]. Also, the MSC-EVs experimental group had improved clot firmness and significantly increased blood clot area. The proteomic analysis demonstrates that both MSCs and MSC-EVs contain several well-known proteins participating in coagulation, such as CD9, PS, myosin-9, talin-1, histones, and cytoplasmic actin. CD9 is one of the most critical proteins in platelet activation initiation, platelet aggregate stability promotion, and fibrinogen binding enhancement. Moreover, umbilical cord MSCs were found to contain TF. However, it was not detected in MSC-EVs. Another exciting finding is that MSC-EVs contain annexin V, a protein characterized by participating in anticoagulant activities. Therefore, it might be speculated that the coagulation properties of EVs depend on pro- and anticoagulant proteins’ dynamic balance [143].

In addition, Chance et al. checked if EVs isolated from three-dimensional cultures have anticoagulant activities associated with the presence of procoagulant activity factors [144]. Scientists determined the procoagulant activity of monolayer and spheroid-cultured AdMSCs and BMSCs-derived EVs (AdMSCs-EVs and BMSC-EVs, respectively). Both EV groups were functionally thrombogenic. They significantly increased the peak of thrombin activity and decreased the time to reach it (*p* < 0.0001). Additionally, the total amount of generated thrombin in all EV groups was markedly increased. Moreover, this study confirmed that the procoagulant activity of EVs is associated with the expression of TF and PS on the surface of vesicles. Such procoagulant activity factors were identified in all the EV groups. However, AdMSC-EVs show greater PS expression, which leads to higher thrombin amounts compared to BMSC-EVs. Also, the strongest clots were formed in the group of treatment with the EVs derived from AdMSCs spheroids. On the other hand, BMSC-EVs demonstrated quicker clot initiation outcomes. In general, it is concluded that all examined EV types have a thrombogenic nature. Another similar study with AdMSC-EVs and BMSC-EVs confirmed that AdMSC-EVs have more significant procoagulant activity in whole human blood or human platelet-poor plasma, which corresponds to the level of TF expression [145]. These data suggest that the anticoagulant properties of MSC-EVs depend on the genes, regulating coagulation, levels of expression, despite cell culturing type.

Typically, proinflammatory cytokines trigger hemostatic activities [146]. Interestingly, AdMSC-EVs have procoagulant activity independent of proinflammatory stimulus [147]. Fiedler et al. investigated EVs from unstimulated AdMSCs and those treated with LPS and TNF proinflammatory substances. Also, clotting experiments were conducted with the EVs compared to reference plasma (a citrated human plasma), coagulation factor XII-deficient plasma, and coagulation factor VII-deficient plasma. In the reference plasma group, all EVs showed similar clotting times. However, in factor VII-deficient plasma, a clot did not form in all EV groups. Due to the absence of clots in factor VII-deficient plasma, authors predicted that EVs might contain TF, which has a role in the activation of VII factor-dependent extrinsic pathway of coagulation. Besides, in factor XII-deficient plasma, unstimulated and TNF-stimulated EVs groups demonstrated significantly increased (*p* < 0.05) clotting time. Prolonged clotting time may indicate that the EVs contain PS molecules, which provide a catalytic surface for factor XII activation. This factor plays an important role in the stimulation of the intrinsic coagulation pathway. Thus, AdMSC-EVs can participate in wound healing via different pathways independent of proinflammatory stimulus [147].

In general, MSC-EVs can maintain wound healing by balancing pro- and anticoagulant molecule supply and affecting blood clot formation pathways and kinetics. The vesicles contain several proteins and lipids, mainly TF and PS, responsible for EV procoagulant activity. In addition, MSC-EVs may participate in hemostasis by activating extrinsic and intrinsic pathways of coagulation. However, there is still a lack of studies indicating MSC-EVs’ role in wound hemostasis.

### 3.2. Mesenchymal Stem Cell-Derived Extracellular Vesicles in Inflammation

Neutrophils clean up the wound site from damaged cells and bacteria. However, macrophages continue the wound bed clearing by phagocytosis of residual tissue and increase the production of cytokines and growth factors, resulting in the enhanced inflammatory response. The key role of anti-inflammatory macrophage properties depends on their ability to switch their phenotype from M1 to M2. A broad range of studies (see Table A2) concluded that macrophages attenuated inflammation with immune modulation by shifting their phenotype after the internalization of stem cell EVs.

Xiaoning et al. checked if EVs isolated from BMSCs stimulated macrophage polarization [148]. In this case, in one of the experimental groups, BMSCs were treated with siRNA, which silenced the expression of the rab27a protein, a regulator of EVs secretion, thus inhibiting EVs release. Compared to the BMSC/siRNA group, macrophages cultured with EVs showed a higher level of M2 macrophages marker—CD206, and this proved the ability of BMSC-EVs to promote macrophage polarization. Furthermore, the EVs’ enhanced cutaneous wound healing in vivo, whereas the rab27a-silenced group had delayed healing. Also, scientists isolated EVs after BMSCs transfection with miRNA-223 mimics and inhibitors. Results indicated that BMSC-EVs, isolated after knockdown of miRNA-223 in BMSCs, reduced macrophage polarization from M1 to M2. Besides, pknox1, miRNA-223 target and regulator of macrophage polarization, gene expression in macrophages was altered, depending on treated BMSC-EVs type. The study revealed that miR-223 is transferred from EVs to macrophages and is responsible for a macrophage phenotype shift [148].

Another study used dermal fibroblasts treated with interferon-gamma (IFNγ) and tumour necrosis factor α (TNFα) as a cellular inflammation model to examine AdMSC-EVs’ anti-inflammatory role in wound healing [149]. Fibroblasts were co-cultured with peripheral blood mononuclear cells. After the addition of AdMSC-EVs, a change in macrophage phenotype from M1 to M2 was observed, demonstrated by a significant increase in expression of Arg1 and CD206, the markers of M2 cells. Moreover, various miRNAs (miR-34a-5p, miR-124-3p, miR-146a-5p) were detected in AdMSC-EVs, which are responsible for macrophage phenotype shift. Besides, the treatment of inflammatory cytokine-stimulated fibroblasts with AdMSC-EVs decreased the expression of inflammatory proteins TNFα, IL-6, and IL-8, while increased the expression of IL-10. Microarray experiments identified several miRNAs (miR-223, miR-203, miR-146a) present in AdMSC-EVs, which participate in various signaling pathways associated with wound healing by targeting factors such as myocyte-specific enhancer factor 2c (Mef2c), TNFα, and anti-inflammatory cytokine—IL-24. Authors hypothesized that the anti-inflammatory effect of AdMSC-EVs was caused by such miRNAs [149].

Liu recently characterized the mechanism of MSC-EV-induced macrophage phenotype change with colleagues [150]. The authors concluded that immunosuppression effects of melatonin-treated BMSC-EVs in diabetic wounds are reached by upregulating PTEN (phosphatase and tensin homolog) expression and inhibiting the phosphorylation of AKT (protein kinase B), i.e., by suppressing PTEN/AKT signaling pathway. Consequently, gene expression of proinflammatory IL-1β, TNFα, and iNOS (M1 macrophage markers) significantly decreased (*p* < 0.05). In contrast, M2 macrophage markers anti-inflammatory IL-10 and Arg1 gene expression raised after the EV treatment. Such EV-mediated balancing of inflammation-related biomolecules might lead to the reduction of prolonged inflammatory periods [150].

In addition, to macrophage phenotype change, AdMSC-EVs also increase (*p* < 0.05) the viability of KCs by suppressing apoptosis. It was shown in the HaCaT cell line after hydrogen peroxide exposure [151]. Treatment with EVs reduced expression of apoptosis-related proteins caspase-3 and IL-6 and elevated expression of inflammation-related biomolecules Bcl-2 and IL-10 (*p* < 0.05). Interestingly, the AdMSC-EVs internalization rate directly correlated with hydrogen peroxide concentration. It was observed that AdMSC-EV treatment increased miRNA-19b levels in HaCaT cells. This miRNA binds to inflammatory factor—chemokine CC motif ligand 1, resulting in activation of the TGF-β pathway, which inhibits the occurrence of inflammation.

Also, it was revealed that the AdMSC-EVs significantly inhibit ROS and inflammatory cytokine expression in EPCs [152]. The vesicles can also improve wound repairing by overexpressing the transcription factor nuclear factor-E2-related factor 2 (Nrf2), which protects cells against oxidative stress [152]. In another study, EVs isolated from umbilical cord MSCs proved their ability to decrease burn-caused inflammation by suppressed NF-κB activation and proinflammatory factors secretion via inhibition of the TLR4 pathway in macrophages [153]. Such findings suggest several new mechanisms of MSC-EVs in suppressing inflammation.

Overall, these data indicate that MSC-EVs can modulate inflammatory processes by changing macrophage phenotype, mainly by transferring miRNAs, resulting in a quicker inflammation stage in wound healing and protection from infections.

### 3.3. Extracellular Vesicles from Mesenchymal Stem Cells in Proliferation

During the proliferation stage, granulation tissue is formed, mainly made of ECM, fibroblasts, inflammatory cells, and blood vessels [154]. Fibroblasts and ECs play a vital role in the formation of this tissue. Also, in the late proliferation phase, the synthesis of a new epidermis occurs. Currently, there are a lot of studies (see Table A2) that indicate the effects of MSC-EVs in cell proliferation and migration activities, angiogenesis, and the epithelization processes during wound healing.

First of all, MSC-EVs affect fibroblast migration to the wound site. Cooper et al. conducted an interesting study measuring electrical current-treated fibroblast migration rate after incubation with AdMSC-EVs [155]. The experimental group treated with the EVs showed a similar fibroblast migration rate compared to the positive control treated with basic FGF (bFGF). In addition, the cellular migration rate value was doubled in the presence of twice higher concentrations of EVs. Besides, the significance of AdMSC-EVs cargo MALAT1 (metastasis-associated lung adenocarcinoma transcript 1, a long non-coding RNA) in maintaining fibroblast migration to wound sites was proved since EVs lacking MALAT1 decreased fibroblast migration rate by halving. Other authors determined that fibroblast migration is supported through downregulation of LATS2 (large tumour suppressor 2) levels by miR-135 transferred by human amnion MSC-EVs [156].

MSC-EVs do not just promote cell migration to the wound site but also improve granulation tissue formation. AdMSC-EVs internalized by fibroblasts increase N-cadherin, cyclin-1, PCNA, collagen I and III gene expression (*p* < 0.001), following in promotion of cell proliferation and inducing collagen synthesis in damaged tissue areas [157]. Another study suggests that AdMSC-EVs affect fibroblast proliferation, increase collagen, bFGF, TGF-β1 gene expression, and protein levels [158]. Additionally, Wang et al. showed that in vitro fetal dermal MSC-EVs promote fibroblast migration and proliferation and increase fibroblast activity detected as an elevation in mRNA expression of collagen, elastin, and fibronectin [159]. Since activated fibroblasts have regenerative effects, the authors investigated that in vivo, in a mouse wound model, the EV-treated group had higher collagen deposition, ECM synthesis, and a faster wound healing rate.

Recently, studies indicated several new MSC-EV cargos participating in proliferation stage activities. Previously described Wang et al. study revealed that after the treatment with EVs, fibroblasts showed increased expression of the components of the Notch pathway, responsible for the regulation of wound-healing-related-cell proliferation and migration [159]. In addition, a ligand of this pathway, Jagged 1, was detected in the EVs. These results determined that MSC-EVs promote fibroblast activity via the Notch signaling pathway by transferring Jagged 1. Qian with colleagues found that AdMSC-EVs accelerate wound healing through long non-coding RNA H19, miR-19b, and SRY-related high-mobility-group box 9 (SOX9) axis [160]. The EVs carried lncRNA H19 that inhibited mir-19b expression and upregulated SOX9, consequently activating the Wnt/β-catenin pathway followed by accelerated fibroblast proliferation, migration, and invasion into the wound bed [160]. Shabbir et al. determined that BMSC-EVs modulate wound healing by inducing the expression of cell cycle progression factors (c-myc, cyclin A1, cyclin D2), growth factors (HGF, IGF1, NGF, SDF1), and cytokines (IL-6) [161]. The authors figured out that MSC-EVs contain STAT3 and can transfer it to recipient cells inducing expression of mentioned genes and activation of signaling cascades, responsible for cell migration, proliferation, and angiogenesis in the wound site. All these findings suggest that EVs participating in different proliferation promoting signaling pathways due to the transferring of multiple cargos to the recipient cells.

It is essential to restore not only granulation tissue structure, but also its function. For this, new blood vessel formation is required. There are some publications indicating MSC-EV importance in new endothelial tube formation due to their proangiogenic activity in wound healing. AdMSC-EVs increase tube length and branches in vitro and in vivo via transferring miR-125a to ECs and inhibiting DLL4 expression [162]. Overexpression of miR-125a upregulated pro-angiogenic (Ang1 and Flk1) genes and downregulated anti-angiogenic (Vash1 and TSP1) gene expression in vitro. Another study investigating immortalized AdMSC line HATMSC1-derived EVs found that they increase proliferation and have proangiogenic properties on human ECs in a dose-dependent manner [163]. The EVs contain growth factors (EGF, bFGF) and pro- and anti-angiogenic factors (IL-8, VEGF, TIMP-1, and TIMP-2), also, several types of miRNAs: proangiogenic (miR-210, miR-296, miR-126, and miR-378) and antiangiogenic (miR-221, miR-222, miR-92a). It was determined that the expression of proangiogenic miRNAs was higher than antiangiogenic ones, resulting in shifting the balance to stimulate angiogenesis. The increased level of miR-296 expression upregulates VEGFR2 in ECs and leads to angiogenesis [163]. In other research, EVs from umbilical cord blood MSCs proved to enhance angiogenesis and accelerate the healing process in a mouse model [164]. The authors studied the expression level of some miRNA in EVs and found that the miR-21-3p was the most intensively expressed. In vitro, this miRNA promotes angiogenic effects by activating PI3K/Akt and ERK 1/2 pathway through the downregulation of miR-21 target genes PTEN and SPRY1 (sprouty homolog 1). Together these data indicate that MSC-EVs can transfer angiogenic signals through miRNAs.

However, angiogenic signals also can be transmitted by other biomolecules. Chun-Yuan Chen et al. analyzed the protein profile of EVs isolated from urine-derived stem cells (USCs) and found that the EVs showed higher levels of angiogenesis promoting protein DMTB 1 compared to the level of the protein in USCs. The study demonstrated the capability of EVs to stimulate angiogenesis through the transfer of DMTB1 protein to ECs [165]. Liu with colleagues determined that human umbilical cord MSC (HUMSC)-derived EVs promote cutaneous wound healing in rats after second-degree burns [166]. Data showed that after such EV application in vivo, the new epidermis was regenerated without a scar, and new vessels were nicely formed in the injury area compared to much worse results in the untreated control. The authors have found that angiopoietins (Ang-1 and Ang-2), the main regulators responsible for vascular maturation, remodeling and stability, were present in the EVs; however, expression of Ang-2 was more significant. Ang-1 participates in vessel stabilization, while Ang-2 regulates the interaction with cell-matrix by binding to integrin in new vessel growing. These biomolecules modulate angiogenesis activities via angiopoietin/TIE signaling pathway [166].

In parallel with new granulation tissue formation and angiogenesis, a new epidermis layer of skin is created. Here, KCs play a central role. First of all, they migrate to the wound edges and proliferate, starting re-epithelization. Experimental data show that MSC-EVs promote the activity of such wound edge KCs. Zhang with colleagues found that AdMSC-EVs support HaCaT cell migration and proliferation in vitro and accelerate wound healing in vivo [167]. They determined that such EVs activate the AKT/HIF-1α pathway, which leads to improved wound healing. Another study demonstrated the signaling is mediated by miR-21 and involves an increase in MMP-9 and TIMP-1 gene expression (*p* < 0.001) [168]. Other signaling pathways stimulated by AdMSC-EVs have also been identified, for example, that of Wnt/β-catenin; Wnt protein promotes nuclear translocation of β-catenin, increasing the expression of this protein, enhancing the proliferation of skin cells. The trigger of the pathway was the Wnt4 protein transferring to skin cells by the EVs, resulting in the increased expression of β-catenin in cultured KCs and stimulation of their migration and proliferation [169,170].

Pomatto et al. conducted an interesting study comparing AdMSC-EV and BMSC-EV activity in wound healing and their cargo content [171]. Both types of MSC-EVs similarly affected fibroblast and KCs migration; however, AdMSC-EVs more effectively stimulated ECs migration and vessel tube formation, while BMSC-EVs were more effective in promoting cell viability and proliferation. The comparison of miRNAs in AdMSC-EVs and BMSC-EVs demonstrated that 14 miRNA is present only in BMSCs-EVs, 70 miRNA—in AdMSC-EVs, and 99 miRNA was detected in both EV types. Based on these data, bioinformatics analysis, using miRpath tool, indicated that the miRNAs from both EV groups are involved in several signaling pathways: the EGFR receptor (ERBB2) signaling pathway triggering previously discussed P13K/Akt downstream signaling cascade; the ECM-receptor interaction, and adherent junction pathways, regulating cell adhesion and migration activities, and the MAPK signaling pathway, controlling cell viability, differentiation and proliferation. The TGF-β and the HIF-1α signaling pathway triggers were indicated in the AdMSC-EVs, but not in the BMSC-EV group. In addition, proteomics analysis revealed 38 proteins present in both EV types, 24 proteins—only in BMSC-EVs, and 41 proteins—in AdMSC-EVs. Panther pathway tool was used for identified significantly correlated pathways for detected proteins. In AdMSC-EVs, the proteins related to angiogenesis were Wnt, FGF, PDGF, TGF-β, and EGF receptor. In BMSC-EVs, detected proteins were linked to cell adhesion (integrin and cadherin) and metabolic processes. Together, these results confirm that EV cargo plays a vital role in maintaining specific biological activity, and occurring cargo differences correlate with the therapeutic effects in different EV types.

In general, all these articles suggest using MSC-EVs as a tool to accelerate wound healing as they participate in all the different proliferative stage processes. First, they stimulate fibroblast migration and proliferation, increasing collagen synthesis and other ECM components, favoring granulation tissue remodeling. Then, on top of that, they promote KCs activity, improving the re-epithelialization process. Finally, EVs also affect ECs, increasing the quantity and maturity of blood vessels and promoting faster wound irrigation.

### 3.4. Extracellular Vesicles from Mesenchymal Stem Cells in Remodelling

It is essential to restore injured tissue in the early healing stages by enhancing collagen production [154]. However, in later tissue remodeling phases, overexpressed collagen could lead to extensive scar formation. Therefore, during the last wound healing phase, tissue maturation takes place when collagen III is replaced with collagen I and dermal appendages (hair follicles, sweat glands) are formed. In addition to stimulation of KC migration and proliferation, MSC-EVs also positively affect tissue maturation and reduce scar formation (see Table A2).

Jiang with colleagues using full-thickness rat skin wound model, have demonstrated that after subcutaneous injection of BMSC-EVs, occurrence of cutaneous appendages, hair follicles, and sebaceous glands in the damaged tissue area significantly increased [172]. The authors noted that such EVs restored normal skin morphology and recovered its function; they significantly elevated the immunostaining intensity levels for α-SMA and VEGF, the critical mediators of angiogenesis and myofibroblasts markers. In addition, the authors found that BMSC-EVs participate in wound healing via inhibiting the TGF-β1/Smad signaling pathway; there were decreased mRNA levels of TGF-β1, Smad2, Smad3, Smad4, and increased those of TGF-β3 and Smad7 levels in the group treated with MSC-EVs compared to PBS control. In general, TGF-β1 is related to fibrosis, while TGF-β3 with anti-fibrotic or scarless activities is related to wound repair. These biomolecules have a vital role in regulating epidermal and dermal cell movement during wound healing. It is even assumed that changes in TGF-β1 and TGF-β3 expression can lead to scarless wound repair. However, another study suggests that AdMSC-EVs induce remodeling of ECM by enhancing the collagen III/collagen I ratio and improving MMP3 expression via protein kinase/mitogen-activated protein kinase (ERK/MAPK) signaling pathway, preventing myofibroblast differentiation to fibroblasts and promoting scarless wound healing [173].

At last, AdMSC-EVs improve skin elasticity and barrier integrity by increasing main skin barrier proteins filaggrin, loricrin, and AQP3 gene expression [174]. Moreover, MSCs-EVs from the umbilical cord and USC-EVs can reduce scar widths [164,165].

In general, MSC-EVs can facilitate tissue remodeling, activating fibroblast differentiation to contractile myofibroblasts and boosting skin appendage formation. Also, they improve skin mechanical properties, such as elasticity and barrier integrity. Finally, MSC-EVs reduce scars and promote scarless wound healing.

## 4. Plant-Derived Extracellular Vesicles

Plant-derived extracellular vesicles (plant derived-EVs) are membranous vesicles that characteristically resemble mammalian exosomes and have a diameter of 40–150 nm. Plant-derived EVs can communicate cross-kingdom (e.g., with mammalian cells), enter the cells, release their cargo, and modulate recipient cell response. In addition, plant-derived EVs, as opposed to mammalian exosomes, do not contain zoonotic or human pathogens [175]. Also, they differ in chemical profiles; their composition contains fewer proteins, and the lipid layer has no cholesterol [176]. Moreover, plant-derived EVs-based therapy strategies would be a safer and more economical alternative as they have lower toxicity, proper tissue-specific targeting, and significant potential for large-scale production [177,178]. Recent studies have investigated plants including grapefruit, grape, ginger, lemon, broccoli [176], and wheat [177], some of which were reported to have promising wound healing properties, summarized in Table A3.

Wound healing is a complex, multi-step process involving various biological responses from different types of cells, secreted mediators, and ECM elements. Throughout human history, plants were used as wound treatment remedies. There is a wide variety of herbs that exhibit wound healing potential. EVs were even found in the xylem and phloem of woody plants [161]. Also, the study on broccoli-derived EVs revealed they could penetrate deep into skin tissue [146]. Thus, plant-derived EVs are a new underexplored field, and they are a promising new biotechnological wound-care agent. A few studies that explored the plant-derived EVs identified the potential in wound healing properties.

### 4.1. Plant-Derived Extracellular Vesicles in Hemostasis

During the hemostasis stage in the wound healing process, blood vessels contract and blood coagulation and clot formation is initiated. Any deviance from the hemostatic balance may lead to health problems. For example, lack of proper clot formation may evoke excessive bleeding. To our current knowledge, there is no data regarding plant-derived EVs’ effect on hemostasis; however, from pharmacognosy and phytochemistry sciences, it is known that some plants have anti-bleeding properties [179]. Also, many examples of hemostatic plants, such as *Rubia cordifolia* (Manjistha) [180], *Ageratum conyzoides*, *Alchornea cordifelia*, *Aspilia africana*, *Baphia nitida*, *Chromolaena odorata*, *Jathropha curcas*, *Landolphia owariensis* are identified in the literature. Additionally, many more plants, for instance, *Aloe spesiosa*, *Beta vulgaris*, *Dalbergia sissoo*, *Humulus lupulus*, *Salix alba,* etc., were tested in vitro and identified as having hemostatic qualities [181]. Most of these plants and their healing properties are known for ages, and their herbal extracts were used in wound healing in traditional medicine [182]. Thus, there are limitless research opportunities in the plant-derived EVs field in hemostasis.

### 4.2. Actual and Predictive Role of Plant-Derived Extracellular Vesicles in Inflammation

The inflammatory stage of wound healing is a necessary natural phase, which may become harmful if prolonged. In this case, a chronic wound may develop, and anti-inflammatory therapeutic strategies should be considered. There are various studies that conducted research on various plant-derived EV effects on different cell lines and animal models, where anti-inflammatory properties were identified. Also, it is known that redox homeostasis is fundamental for proper wound healing, and plant-derived EVs were shown to modulate this balance. Even though a small amount of ROS is necessary for proper wound healing, the excess of ROS and reactive nitrogen species (RNS) leads to oxidative stress, which impairs wound repair and is thought to be related to chronic and non-healing wounds. Thus, modulation of anti-oxidant properties may be an essential strategy in the inflammatory wound healing stage [41].

Nicola Baldini et al. showed that nanovesicles derived from Citrus limon L. juice contain citrate, vitamin C, and short RNA sequences (20–30 bp). The incubation of human mesenchymal stromal cells with these plant-derived EVs in vitro resulted in EV uptake by the cells and the significant protective effect against oxidative stress. It is speculated that this may be due to the direct delivery of micronutrients that are well preserved inside the nanovesicle [183]. Likewise, Francesca Perut et al. demonstrated similar antioxidant effects of strawberry juice-derived EVs on mesenchymal stromal cells in a dose-dependent manner [184]. Additionally, blueberry-derived EVs reduced (*p* < 0.01) oxidative stress in rotenone-stimulated HepG2 cells and high-fat diet-fed C57BL/6 mice. After incubating rotenone-treated HepG2 cells with the blueberry-derived EVs, the level of ROS was decreased, mitochondrial membrane potential was increased, and cell apoptosis was prevented. The effects were mediated by stimulating the expression of Bcl-2 and heme oxygenase-1 and reducing the content of Bax. Also, the translocation of Nrf2, a critical transcription factor of antioxidative proteins, occurred from the cytoplasm to the nucleus in rotenone-treated HepG2 cells. In addition, the EVs increased the expression of antioxidant genes in hepatocytes of high-fat diet (HFD)-fed mice. Furthermore, the expression of two key transcription factors for de novo lipogenesis in the liver of HFD-fed mice was inhibited [185]. In another study conducted by Mariangela de Robertis et al., cellular uptake of blueberry-derived EVs was investigated on the EA.hy926 ECs line, and the protective effect against TNF-α-induced inflammatory gene expression and ROS generation was demonstrated [186].

Grape-derived EVs were shown to have protective effects against dextran sulfate sodium-induced colitis and mediate intestinal tissue remodeling [187]. Similarly, colitis was reduced, and intestinal wound repair was promoted by *Curcuma Longa*-derived EVs in the mice model [188]. Next to ginger and grapefruit EVs, anti-inflammatory and anti-oxidative properties on intestinal health and activation of Wnt signaling of carrot-derived EVs was shown in the study conducted by Jingyao Mu et al. [189]. EV-mediated lung inflammation was counteracted with ginger-derived EVs, which revealed anti-inflammatory therapeutic potential [190]. Thus, there is mounting evidence for plant-derived EVs as playing a role in the inflammatory wound healing stage, but more direct research is required to reveal their entire mechanism of action.

### 4.3. Plant-Derived Extracellular Vesicles in Proliferation

Proliferation follows and overlaps with the inflammation stage. During this phase, re-epithelization and angiogenesis occur and granulation tissue is formed [191]. Grapefruits` EVs in a dose-dependent manner increased HaCaT cells’ viability and cell migration and reduced intracellular ROS production. Additionally, treatment of HUVECs with grapefruit-derived EVs increased the tube formation capabilities [192]. *Triticum aestivum*, or in other words, common wheat, extracts are often used in traditional medicine for their natural healing properties. Wheatgrass juice–derived EVs significantly increased viability and migration of endothelial, epithelial, and dermal fibroblast cells in a dose-dependent manner, enhancing wound closure. Moreover, the EVs had an angiogenic effect stimulating ECs to increase vascularization and promote wound healing [177]. Ginger-derived EVs also induce intestinal wound healing by reducing the expression of hemopexin and altering the expression of other mitochondrial and cytoplasmic proteins such as heat shock protein, axin, and kinesin [176,193]. Currently, ginger-derived EVs with and without curcumin are being explored in inflammatory bowel disease in a clinical trial, which is in recruiting status (NCT04879810). Also, ginger-derived EVs are tested in a clinical trial for efficiency against colon cancer (NCT01294072). Even though ginger-derived EVs have reached the clinical trial stage, there is much unknown regarding various plant EV roles and effects in the proliferation phase so far.

### 4.4. Plant-Derived Extracellular Vesicles in Remodelling

During this last stage, which is also known as maturation, the scar is formed. Collagen is remodeled from type III to type I, and the wound fully closes. Also, cross-linking of collagen and apoptosis of unnecessary cells occurs [194]. However, there is no data regarding plant-derived EVs on wound remodeling and scarring. Still, several herbal extracts and active herbal compounds have been shown to reduce hypertrophic scar and keloid formation, such as onion extract, grapes, and peanut-derived resveratrol, epigallocatechin gallate from green tea and others [195]. Future research on whether EVs derived from these or other plants play a role in tissue remodeling in wound healing might provide new insights and potential therapeutic opportunities.

## 5. Therapeutical Application of Extracellular Vesicles for Skin Wound Healing

In the last few years, interest in MSC-derived EVs as a therapeutic tool has increased in regenerative medicine [138]. Recent studies showed promising applications of such EVs due to their cargo specificity, built on EVs’ secreted cell origin [196]. These nanovesicles can go through various biological barriers, including the blood-brain barrier; further, their cargo is well preserved and protected from degradation [197]. In comparison with stem cell therapy, EVs reduce the risk of immunogenicity, tumorigenesis, avoid cell differentiation to unexpected derivation. Moreover, it is possible to employ their cargo and achieve desired therapeutic effects [198]. All these properties are essential for considering EVs to maintain tissue regeneration processes.

### 5.1. Extracellular Vesicle-Loaded Scaffolds

Wound healing is a complex, dynamic, and highly regulated physiological process involving orchestrated activation of signaling pathways and molecular mechanisms to regenerate tissue microenvironment consisting of various cell types. To support the healing process, wound dressings are used to cover the wound and to provide protection against infection and mechanical stress, and promote tissue regeneration. Traditionally used wound dressings such as bandages, gauzes, and cotton are not effective enough as they fail to maintain an optimal level of moisture essential for wound healing, collagen synthesis and angiogenesis [199]. Due to advancements in regenerative medicine, modern wound dressings are made using biomaterials such as natural or synthetic polymers forming three-dimensional structures known as scaffolds. Biologically active compounds, cells or nanoparticles can be incorporated into the scaffolds creating bioactive wound dressings with improved biocompatibility and bioactivity [199,200]. Cell-derived EVs have a significant role in wound healing by mediating every step of this process [138]. Despite many advantages of using EVs in wound healing, there are some challenges. It is challenging to keep them in target sites due to their ability to travel far away from the application site. Also, topically applied EVs can have rapid clearance by fluids` and may be damaged by external factors [138,201]. To keep EVs at the wound site and promote longer-lasting and more efficient results of wound healing, they can be encapsulated into scaffolds.

The most attractive scaffolds for tissue regeneration are hydrogels. They are porous, hydrophilic polymeric structures obtained by physical or chemical cross-linking [201]. Ideal scaffolds should have the ability to absorb wound exudate without being dissolved. The porous structure is also needed to ensure a proper vascularization process, to maintain homeostasis and keep the normal tissue temperature and gas exchange at the wound site. Adhesiveness and biodegradability are other advantageous traits of ideal scaffolds [199,202]. Hydrogels usually consist of natural polymers, for instance, alginate, chitosan, collagen, and therefore, they are biocompatible with the natural tissues. In addition, these polymers have specific biological functions in wound healing. For instance, chitosan is a natural polysaccharide known to enhance the granulation of tissue formation, promote adsorption of fibrinogen and platelet adhesion, as well as collagen and hyaluronic acid formation [199,203]. Alginate, another natural polysaccharide used for scaffold formation, can stimulate cytokine production with monocytes. Collagen and its derivative gelatin, when used as a scaffold, facilitate cell adhesion and proliferation and provide structural support for connective tissue [199]. Synthetic polymers, such as polyethene glycol (PEG), poly(glycolic acid) (PGA), polyurethane (PU), or a combination of natural and synthetic ones, can also be used [202]. Biocompatible hydrogels are applied for the non-invasive and straightforward delivery of large amounts of EVs to the target site [138]. EVs can be encapsulated in hydrogels in three different ways: by mixing with polymers before the addition of cross-linking agents; physically added after hydrogel polymerization; used with polymers and crosslinkers at the same time during in situ mixings [201]. Examples of different types of EV-loaded scaffolds that may be used for wound healing are summarized in Table 1.

Nooshabadi et al. developed a chitosan-glycerol hydrogel loaded with EVs isolated from human endometrial stem cells (hEnSC) [204]. In vitro studies showed that EVs and CTF-glycerol hydrogels have synergistic effects on human skin fibroblast cell proliferation. In vivo experiments with mouse full-thickness excisional wound model resulted in a maximum level of fibrosis, vascularization, and epithelial thickness after treatment with CTF-glycerol-Exo hydrogel compared to control groups (CTF hydrogel and sterile paraffin gauze). Observed effects are attributed to hEnSC-derived EVs as they contain biomolecules such as growth factors VEGF, bFGF, and TGF-β1, which are involved in angiogenesis. Thus, the CTF-glycerol-Exo hydrogel could be used as an efficient scaffold for skin regeneration and wound healing.

One of the main functions of wound dressings is to protect the wound from bacterial infections, which negatively affect the wound healing process [200]. To combat this medical threat, Qian et al. produced an asymmetric composite made of wettable chitosan-silk fibroin/sodium alginate wound dressing with embedded silver nanoparticles (AgNPs) and EVs isolated from HUMSCs [205]. Silver nanoparticles are known to have broad-spectrum antibacterial activity and are often used in the clinic. It was revealed that the asymmetric modification of the dressing created two different surfaces: one hydrophilic and porous, and the other hydrophobic and smooth. This approach is beneficial for wound healing as the hydrophilic side can absorb excess fluids, maintain electrolyte balance, and release AgNPs-EVs over time, while the hydrophilic surface provides a physical barrier and protects against microorganisms. In vivo studies with *Pseudomonas aeruginosa*-infected full-thickness wound mouse model demonstrated that after seven-day treatment, new vessel formation was increased as evidenced by elevated expression of CD34 and alpha-smooth muscle actin (*p* < 0.05). Moreover, the dressing exhibited an antibacterial effect and increased cell proliferation. All these results indicate that CTS-SF/SA/AgNPs-EV wound dressings could be used to promote the healing of infected wounds.

Wang et al. developed methylcellulose-chitosan hydrogel (MC-CS) with embedded EVs isolated from placental MSCs (PMSC) [206]. Experiments with diabetic mouse full-thickness wound model revealed that ten-day treatment with the MC-CS-EV hydrogel promoted the activation of fibroblasts and KCs, accompanied by the formation of nerves and sweat glands. Moreover, results indicated faster re-epithelialization, angiogenesis, and inhibition of apoptosis. Thus, MC-CS-EV hydrogel can promote wound healing and recover skin structure by stimulating new tissue and vessel formation in a diabetic mouse model.

Another research group led by Zhao et al. developed gelatin methacryloyl (GelMA) hydrogel with encapsulated EVs from HUVECs [207]. In vitro studies with KCs and fibroblasts revealed HUVECs-EVs’ ability to accelerate the proliferation and migration of these cells. In vivo studies demonstrated that GelMA with HUVECs-EVs treatment accelerated wound re-epithelialization and angiogenesis. Moreover, after 14-day therapy, the wound with GelMA hydrogel with encapsulated HUVECs-EVs showed increased collagen deposition and collagen III and I expression (*p* < 0.05). Based on results, the GelMA wound dressing can provide sustained release of HUVECs-EVs and be used for wound repair.

Shiekh et al. created OxOBand wound dressing composed of antioxidant polyurethane and EVs isolated from AdMSCs [208]. In vitro experiments demonstrated Ad-SC-EVs capability to increase attachment, proliferation and migration rate of KCs and fibroblasts and reduce oxidative stress. In addition, studies using diabetic rat models showed accelerated wound closure, re-epithelialization, granulation tissue formation, angiogenesis, and collagen remodeling after treatment with OxOBand.

Despite hydrogels being one of the most commonly used scaffolds, other biomaterials can be used for wound dressings. Decellularized biomaterials are gaining interest in regenerative medicine. These biomaterials are obtained by the decellularization process where cells of living tissue are chemically or physically separated, creating an acellular ECM scaffold. One example of such a scaffold used for tissue and organ repair is the human acellular amniotic membrane (hAAM). Xiao et al. constructed a combination of hAAM with AdMSC-derived EVs [209]. In vitro, AdMSC-EVs promoted proliferation of HUVECs and human dermal fibroblast (HDF) cells and stimulated tube-forming ability of HUVECs and migration of HDFs. In vivo studies carried out in diabetic mouse models revealed that hAAM-EVs’ wound dressing accelerated wound closure, vascularization, collagen deposition, and regeneration of skin appendages such as hair follicles and sebaceous glands. To summarize, a scaffold made from hAAM is suitable as a delivery system for EVs to treat diabetic wounds.

### 5.2. Application of Extracellular Vesicles for Treatment of Dermal Diseases

EVs from different sources have shown various effects and potential in other dermal applications than wound healing. For instance, AdMSC-EVs have the ability to alleviate atopic dermatitis. Such nanovesicles enhance stratum corneum hydration, de novo ceramides synthesis and significantly reduce inflammatory cytokines (IL-4, IL-5, IL-13, TNF-α, IFN-γ, IL-17) secretion when applied in vivo [11]. Additionally, AdMSC-EVs were identified as capable of participating in skin brightening. This effect was tested with a cosmetic product containing AdMSCs-EVs in a prospective, split-face, double-blind, randomized placebo-controlled study with human volunteers with hyperpigmentation [210]. Results showed that the EVs reduce intracellular melanin levels and, as a result, cause a skin brightening effect. Besides, AdMSC-EVs alleviate photo-ageing by reducing ROS, MMP production, and collagen degradation [211].

Next to their skin health modulating properties, the EVs also have benefits in hair regrowth. A new strategy for androgenetic alopecia treatment to restore the hair follicles cycle is considered. MSC-EVs contain various growth factors and cytokines, which can be involved in hair regeneration [212]. They promote *de novo* morphogenesis of hair follicles and the dermal papilla formation, a vital component modulating follicle cell activity in hair regeneration via Wnt signaling [213]. TGF signaling cascades lead to hair follicle down-growth and hair follicle shape development [214]. Stem cell-secreted EVs also modulate hair re-growth through Fgf, Bmp, Shh, Notch signaling pathways, followed by dermal condensate (accumulation of stem cells) formation, activation and promotion of placode formation (spaced thickenings in the epidermis), and differentiation of hair shaft [212].

Regarding plant-derived EVs, a nanoparticle composition containing ginseng, pine tree leaf, *Salvia militiorrhiza,* and other plant-derived EVs were shown to have hair regrowth promoting effect by deeply penetrating the skin and providing nutrients, stimulating hair follicles and exerting anti-oxidant activity on the scalp [215]. Although not much research has been done regarding hair regrowth and plant EVs, the horizons are open for exploring the wide range of plants known to have hair growth modulating properties [216].

## 6. Future Perspectives

**Figure 8:**
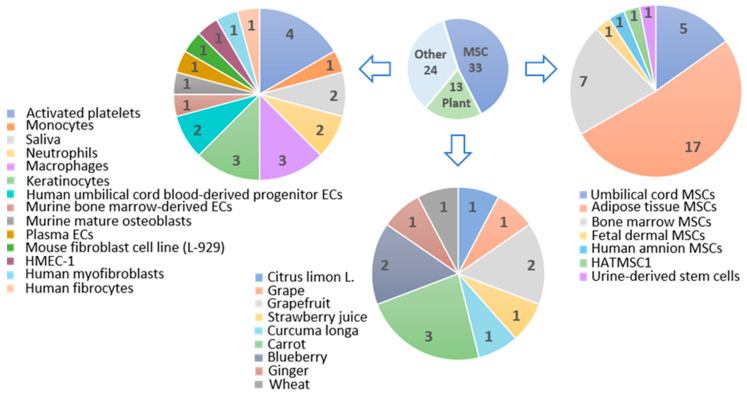
The number of experimental research studies discussed in this review, distributed according to the EV source. MSC—mesenchymal stem cells, EC—endothelial cells, HMEC-1—human microvascular endothelial cell line, HATMSC1—immortalized adipose MSC line.

According to the resources discussed in this review, the most solid amount of experimental data on EV involvement in skin wound healing is about MSC-EVs, with AdMSC-EV leading among them (Figure 8). The role of EVs from activated platelets, neutrophils, and macrophages also attracts the interest of scientists, followed by that of keratinocytes.

Considering the future insights, besides further investigation of EVs from the less studied or unstudied sources, the EVs can be used as drug delivery matrices for the encapsulation of therapeutic agents, taking advantage of their biocompatibility. AdMSC-EVs loaded with miR-21-5p, a therapeutic candidate for diabetic wound healing, promote in vitro KCs proliferation and migration; in vivo increased re-epithelialization, formation and maturation of vessels and collagen remodeling [217]. In another study, EVs were derived from Human Embryonic Kidney (HEK293) cells and were engineered to contain miR-31-5p. It was shown that these designed EVs promoted healing of diabetic wounds by enhancing angiogenesis, fibrogenesis, and re-epithelization [218]. Thus, engineered designer EVs hold potential for drug delivery and cell-free applications in the wound healing treatment. However, although EV efficacy has been proven, the underlying mechanism is not fully clear. Further research is needed for more efficient AdMSC-EVs products and applications in clinical practice [219].

Regarding plant-derived EVs, they hold a high potential as drug delivery agents, as their use is safe and cost-effective. They provide stability and solubility while also not modifying the biological activity of a loaded cargo. In addition, plant-derived EVs can be harvested in large amounts, which makes them an attractive drug delivery system [220,221]. However, plant-derived EVs are poorly investigated compared to animal and human cell-derived ones, as reflected in Figure 8’s central and lower charts. The high heterogeneity of plant-derived EVs population and lack of specific markers make their characterization a challenge. Comprehensive omics analysis might help to identify potential characteristics and molecular profiles of EVs from different species [222]. Furthermore, future protocols with certain stimuli for specific plants species might be developed to produce naturally derived EVs containing qualities of interest, such as wound healing properties.

Both engineered and naturally derived EVs encapsulated in hydrogels are promising techniques for wound healing because they deliver EVs in the wound site and ensure prolonged and sustained release [223]. The scaffolding technologies are moving towards developing so-called smart hydrogels that can sense and respond to wound environmental parameters, such as pH, ROS levels, glucose concentrations, and so on [224]. By improving some properties of liposome-loaded and EV-loaded hydrogels, the release of these nanovesicles can be better controlled [225]. In response to specific stimulation (near-infrared laser irradiation or pH), substances in the scaffold are delivered in a tuned manner without instant explosive drug release. Future direction should be precise gradual administration of desired EVs at different stages of wound healing [224]. It has been shown that the combination of nanomedicine and oxygen-producing materials (such as SPO, CaO_2_, MgO_2_, H_2_O_2_) relieve wound hypoxia and improve chronic diabetic wound healing [226,227]. The next step might be developing new multi-functional biomaterials (e.g., improving hypoxia, enhancing angiogenesis, reducing oxidative stress) that would provide a balanced environment and regulate wound healing at all stages [224]. What is more, there is no data regarding smart hybrid nanovesicles (liposome and EVs hybrids) and their coupling with hydrogel systems [225]. Overall, self-healing injectable smart hydrogels combined with the smart designer EVs, either of mammalian or plant origin, might be the remedy in the wound treatment future.

## Figures and Tables

**Table 1:** EV-loaded scaffolds and their therapeutic effects in wound healing.

	Scaffold Materials	Scaffold Formation and EV Loading Method	EVs Source	Evs Characteristics		Therapeutic Effects	References
				Size	Surface Marker		
Mouse full-thickness excisional wound model	Chitosan-glycerol hydrogel	Electrostatic interaction between chitosan and glycerol groups; hydrogen-bonding interactions between the chitosan chains. EVs were mixed in to the scaffold mixture	Human endometrial stem cell (hEnSC)	40–150 nm	CD63	↑ angiogenesis, epidermal layer and tissue granulation formation	[204]
Mouse infected full-thickness wound model	Chitosan—silk fibroin/sodium alginate (CTS-SF/SA) dressing with incorporated silver nanoparticle-EVs composites (AgNPs-EVs)	Lyophilized CTS-SF rehydrated, frozen and SA solution added on the surface of the CTS-SF dressing. AgNPs-EVs mixture was prepared by sonication and integrated into CTS-SF/SA by secondary freeze-drying	Human umbilical cord MSCs	30–70 nm	No data	Broad-spectrum antimicrobial activity, ↑angiogenesis, collagen deposition and nerve repair, oxygen and nutrient transfer to the wound was maintained due to moisture retention feature of the dressing	[205]
Diabetic mouse full-thickness wound model	Methylcellulose-chitosan hydrogel	Hydrogel was prepared by one pot mixing of aldehyde modified methyl-cellulose, chitosan grafted poly(ethylene glycol) and EVs. Self-healing properties of the hydrogel determined by dynamic Schiff base linkages between aldehyde and amino groups	Placental MSCs	About 62.5 nm	CD9, CD63, CD81	↑ migration of fibroblasts and KCs and other cells, angiogenesis, re-epithelialization. Inhibition of apoptosis	[206]
Full-thickness cutaneous wound model	Gelatin methacryloyl (GelMA) hydrogel	GelMA was made by reaction between gelatin and methacrylic anhydride. The polymer was dialyzed and freeze-dried. EVs were incorporated by ultraviolet light-induced crosslinking	HUVECs	50–140 nm	CD9, CD63, CD81, HSP70	↑ wound healing, angiogenesis, collagen deposition, re-epithelialization, migration and proliferation of KCs and fibroblasts	[207]
Diabetic rat wound model	Polyurethane-based oxygen releasing antioxidant scaffold (PUAO-CPO)	PUAO-CPO was made by synthesis of PUAO via addition of ascorbic acid to the backbone chain of polyurethane, and subsequent incorporation of calcium peroxide into PUAO cryogels. EVs were attached by incubation forming OxOBand wound dressing (PUAO-CPO-EXO)	AdMSCs	100–300 nm	CD81	↑ vascularization, ↑ KCs and fibroblast migration, proliferation, ↑ collagen remodeling, ↓ oxidative stress	[208]
Diabetic mouse skin wound model	Human acellular amniotic membrane (hAAM)	Decellularization of amniotic tissue	AdMSCs	47.7–150 nm	CD9, CD81	↑ wound healing ↑ vascularization, ↑ ECM production, collagen deposition	[209]

## Data Availability

Not applicable.

## Appendix A

**Table A1:** The role of extracellular vesicles in different wound healing phases.

Phase	Parental Cells	Recipient Cells	Effects	Cargo/Signaling Pathway	Reference
Hemostasis	Thrombin activated platelets	Platelets	Formation of fibrin in vitro ↑, bleeding time and blood loss in vivo ↓	Activated form of integrin αIIbβ3	[103]
	ADP activated platelets	Plasma	Formation of fibrin ↑; Provide pro-coagulant surface and bind PSGL-1	PS, P-selectin	[104]
	Sepsis activated platelets	Plasma	Formation of thrombin ↑; Activate intrinsic and extrinsic coagulation pathways.	PS, Factor XII and TF.	[106]
	Monocytes	Collagen-activated platelets	Transfer of molecules to platelet membrane	TF, PSGL-1	[109]
	Saliva	Plasma	Formation of fibrin ↑; Activate TF-dependent coagulation pathway.	CD24, TF	[110]
	CRP or TRAP-6 activated platelets	Platelets	Platelet activation ↑; Generate superoxide and activate platelets via GPVI receptor, increase P-selectin exposure.	NOX-1	[111]
Inflammation	Opsonized zymosan A activated neutrophils	Neutrophils, HUVECs	↑ ROS, IL-8; E-selectin, VCAM-1	Not determined	[112]
	Resting state neutrophils	Neutrophils, HUVECs, plasma	↓ ROS, IL-8, ↑ Coagulation	Not determined	[112]
	Apoptosing neutrophils	Plasma	↑ Coagulation	Not determined	[112]
	Non-adherent fMLF activated neutrophils	EVs alone HUVECs	↑ ROS, ↑LTB4 Migrated towards chemotactic gradient ↓ *STAT1*, *NFKBIZ*, *CCL8*, *CXCL6*	NOX-2 LKHA4 5-LOX	[113]
	Adherent fMLF activated neutrophils	EVs alone HUVECs	↑ LTB4; Migrated towards chemotactic gradient *↑**IL1**β*, *CCL3L1*, *STAT3*	Not determined	[113]
	Bone marrow derived-IL-4 activated—M2 macrophages	Bone marrow derived-IFN-γ activated—M1 macrophages In vivo healthy mouse model	Macrophage reprogramming M1 → M2 ↓iNOS, ↑Arg1; Reprogrammed M2 ↑ fibroblast migration, EC tube formation; In vivo: ↑ wound healing	CCL24, CCL22, MFG-E8	[117]
	Mouse wound edge KCs	EVs alone; Mouse wound macrophages (M) In vivo healthy mouse model	Glycan ions with high mannose, ↑M uptake; M reprogramming ↓NOS2, CD74, TNFα; ↑ CL3; In vivo: ↓accumulation of M; ↓iNOS, ↑Arg1; ↑Skin barrier-function	Not determined	[7]
Proliferation	Human umbilical cord blood derived endothelial progenitor cells	HMEC-1 In vivo diabetic rat model	↑ Proliferation, migration, tube formation. ↑ *ANG-1*, *E-selectin*, *FGF1*, *CXCL16*, *eNOS*, *VEGFA*, *VEGFR-2*, *IL8*; ↓*MMP-9*. In vivo: ↑ Wound healing	Not determined	[118]
			↑ Proliferation, migration, tube formation. ↑ *FGF-1*, *IL8*, *IL6*, *VEGFA*, COX-2; c-Myc, Id1, pERK1/2 expression gene and protein expression; In vivo: ↑ Wound healing, angiogenesis	miR-21; Activated ERK1/2	[119]
	Murine bone marrow-derived ECs	In vivo healthy and diabetic mice	↑ skin wound healing; ↑ VEGF, PECAM-1, Ki67.	miR-221-3p	[120]
	Macrophages (RAW 264.7)	Mouse endothelial cell line SVEC4-10EHR1; In vivo healthy mouse model	↑ Proliferation, migration, tube formation In vivo: ↑ Angiogenesis	VEGF, Wnt3a, miR-130a, miR-126, miR-210.	[122]
		Diabetic HUVECs In vivo diabetic rat model	↑Angiogenesis, cell migration, proliferation via ↓ IL-6 and TNF-α production; In vivo: ↓ IL-6, TNF-α; ↑ p-Akt, ↓MMP-9; ↑collagen deposition	Activated Akt/VEGF	[121]
	Murine mature osteoblasts	Brain-derived endothelial cell line (bEnd.3)	↑ Proliferation, migration, tube formation; ↑ p-VEGFR2, pERK1/2 expression, MMP-2	MMP-2 Activated:VEGF/ERK1/2	[123]
	Saliva	HUVECs In vivo healthy mice	↑ Proliferation, migration, tube formation; ↓SMAD-6; ↑BMP2; In vivo: ↑ wound healing	UBE2O mRNA	[124]
	Plasma ECs	Diabetic skin fibroblasts; HaCaT; In vivo diabetic mouse model	↑Proliferation, migration; ↓ Senescence markers; ↑ YAP dephosphorylation and nuclear translocation In vivo: ↑ wound healing, ↓fibroblast senescence	Activated PI3K/Akt/mTOR	[125]
	HaCaT; HEKa; NHEK	Human dermal fibroblasts	↑ *TGFBRII*, *CCN2*, *FGF2; laminin-111*, *collagen IV*, *IL-8*, *MMP-1*, *IL6* ↑ IL-6; MMP-1; MMP-3; THBS protein expression; ↑migration, fibroblast-mediated endothelial tube formation. ↓*TIMP3; TIMP4;*	Activated: ERK1/2, Smad, p38; JNK.	[127]
	HEKa	Human foreskin fibroblasts In vivo diabetic rat model	↑migration, fibroblast-mediated endothelial tube formation. ↑ IL-6, IL-8 gene and protein expression; ↓PTEN, RECK; ↑ α-SMA and N-cadherin; ↑ pERK1/2 In vivo: ↑ wound healing.	miR-21; Activated: ERK1/2	[128]
Remodeling	Mouse fibroblast cell line (L-929)	Mouse fibroblast cell line (L-929); mouse endothelial cell line SVEC4-10EHR1; In vivo healthy mouse model	*↑**Collagen I*, *MMP-1*, *MMP-3;* ↑ Proliferation, migration, tube formation; Combination with fibrin glue ↑ wound healing, collagen deposition; *↑* VEGF	Not determined	[130]
	HMEC-1 under hypoxic conditions	ECM	*↑*LOX activity; *↑*Collagen gel contraction	LOXL-2	[131]
	Human myofibroblasts from normal skin wound	Human skin fibroblasts	↑ Migration, collagen I;	PLGF-1, LTA, VEGF, IL-23	[133]
	Human fibrocytes stimulated with PDGF, TGF-β, FGF-1	Diabetic human ECs, KCs, dermal fibroblasts; In vivo diabetic mouse model	↑ Proliferation, migration, tube formation; ↑Collagen I, Collagen III, α-SMA in fibroblasts In vivo: ↑ wound healing.	HSP-90α, total and activated STAT3, miR-124a, miR-125b, miR-126, miR-130a, miR-132, miR-21	[134]

Abbreviations: 5-LOX-5-lipoxygenase, ADP-adenosine diphosphate, ANG-angiogenin, Arg-arginase, bEnd3-brain-derived endothelial cell line, BMP2-bone morphogenic protein 2, CCL3L1-(C-C motif chemokine ligand 3 like 1, CCL-chemokine CC motif ligand, c-myc-cellular myelocytomatosis oncogene, COX-cyclooxygenase, CRP-collagen-related peptide, CXCL-chemokine (C-X-C motif) ligand, ECs-endothelial cells, KCs-keratinocytes, ECM-extracellular matrix, eNOS-endothelial nitric oxide synthase, ERK-extracellular signal-regulated kinase, EVs-extracellular vesicles, FGF-fibroblast growth factor, GPVI-glycoprotein VI (platelet) receptor, HEKa-normal adult epidermal keratinocytes; HMEC-1-human microvascular endothelial cell line, HSP-90α-heat shock protein HSP 90-alpha, HUVEC-human umbilical vein endothelial cells, Id1-DNA-binding protein inhibitor, IFN—interferon, IL-interleukin, iNOS-inducible nitric oxide synthase, JNK-c-Jun N-terminal kinase, Ki67-proliferation marker protein, LKHA4-leukotriene A4 hydrolase, LTA-lymphotoxin alpha/tumour necrosis beta, LTB4-leukotriene B4, miR-microRNA, M1-pro-inflammatory macrophage, M2-anti-inflammatory macrophage, MFG-E8-milk fat globule epidermal growth factor 8, M-macrophage, NFKBIZ-NF-kappa-B inhibitor zeta, NHEK-newborn epidermal keratinocytes, NOS2-nitric oxide synthase, NOX–NADPH oxidase, p-Akt-phosphorylated protein kinase B, PECAM-platelet endothelial cell adhesion molecule-1, PLGF-placental growth factor, PSGL-P-selectin glycoprotein ligand, PS-phosphatidylserine, PTEN-phosphatase and tensin homolog, RECK-reversion-inducing cysteine-rich protein with Kazal motifs, ROSreactive oxygen species, SMAD6-mothers against decapentaplegic homolog 6, STAT-signal transducer and activator of transcription, SVEC4-10EHR1-mouse endothelial cell line, TF-tissue factor, TGFBRII-transmembrane serine/threonine kinase, THBS1-thrombospondin 1, TIMP-metalloproteinase inhibitor, TNF-tumour necrosis factor, TRAP-6-thrombin receptor activator peptide 6, UBE2O-ubiquitin-conj ugated enzyme E2O, VCAM-vascular cell adhesion molecule, VEGFA-vascular endothelial growth factor A, VEGFR-vascular endothelial growth factor receptor, VEGF-vascular growth factor, PSGL-1—P-selectin glycoprotein ligand-1, Wnt-wingless, YAP-Yes-associated protein, αIIbβ3-integrin alpha IIb/β3, α-SMA-α-smooth muscle actin. Italic font means changes in gene expression.

**Table A2:** The role of mesenchymal stem cell extracellular vesicles in wound healing.

Phase	EVs Source	Effects	EVs Molecules Involved	Signaling Pathway	Reference
Hemostasis	Umbilical cord MSCs	Coagulation activation ↑Clot firmness and area ↓Clotting time ↓Clot formation ↓Lag period of spontaneous clotting	PS, CD9, Histones, Myosin-9, Talin-1, cytoplasmic 1 and 2 actin, annexin V	Not specified	[143]
	Adipose tissue MSCs Bone marrow MSCs	↑Peak of thrombin activity ↑Thrombin generation ↓Thrombin activation times Tendency to faster clot formation	PS, TF	Not specified	[144]
	Adipose tissue MSCs Bone marrow MSCs	Procoagulant activity	TF	Not specified	[145]
	Adipose tissue MSCs	Procoagulant activity	Not determined	Extrinsic and intrinsic	[147]
Inflammation	Bone marrow MSCs	Macrophage polarization and reprogrammation M1→M2 ↑CD206 M2 marker ↑*IL-10* and ↓*TNFα* In vivo: ↑Wound closure	miR-223	*pknox1* regulation	[148]
	Adipose tissue MSCs	Macrophages polarization M1 → M2 ↑*Arg1* and ↑*CD206* M2 markers ↓*TNFα*, ↓*IL-6*, ↓*IL-8* and ↑*IL-10*, ↑*TGFβ1*, ↑*TSG6*, ↑*collagen III* and *I*, ↑*fibrinonectin* *In vitro*: ↓Wound area	miR-34a-5p, miR-124-3p miR-146a-5p, miR-132 miR-21, miR-29a miR-223-3p, miR-203b-5p	Notch1 Mef2c Targeting TNFα, IL-24	[149]
	Bone marrow MSCs	↓*IL-1β*, ↓*TNFα*, ↓*iNOS*, ↑*IL-10*, ↑*Arg1* ↑*PTEN*, inhibiting p-AKT, ↑M2/M1 ratio In vivo: ↑Angiogenesis and ↑collagen synthesis	Not determined	PTEN/AKT	[150]
	Adipose tissue MSCs	↑Bcl-2 and ↑IL10 ↓C-caspase3 and ↓IL-6 ↑Cell viability, ↓apoptosis ↑ KCs migration	miRNA-19b	CCL1/TGF-β	[151]
	Adipose tissue MSCs	↓ROS, ↓NOX1, ↓NOX4 ↓IL-1β, ↓TNFα, ↓IL-6 ↑SMP30, ↑VEGF, ↑p-VEGFR2	Nrf2	Nrf2 overexpression	[152]
	Umbilical cord MSCs	↓NF-κB activation ↓IL-1β, ↓TNFα and ↓IL-10	miR-181c	TLR4	[153]
Proliferation	Adipose tissue MSCs	↑Fibroblast migration ↑Angiogenesis In vivo: ↓ischemic wounds	MALAT1	Not specified	[155]
	Human amnion MSCs	↑Fibroblast migration, proliferation ↓E-Cadherin, ↓N-Cadherin, ↓LATS2, ↑αSMA In vivo: ↑Wound healing, new granulation tissue, ↓inflammatory cells amount	miR-135a	LATS2	[156]
	Adipose tissue MSCs	↑Fibroblast migration and proliferation, ↑*N-cadherin*, ↑*cyclin-1*, ↑*PCNA*, ↑*collagen I* and *III* In vivo: ↑Cutaneous wound healing and ↑collagen synthesis	Not determined	Not specified	[157]
	Adipose tissue MSCs	↑Fibroblast migration and proliferation, ↑*collagen I* and *III*, *↑MMP1*, *↑bFGF*, *↑TGF-**β1*, ↑p-Akt/Akt, ↑collagen I and III, ↑MMP1, ↑bFGF, ↑TGF-β1. *In vitro* and *in vivo*: promote and optimize collagen deposition	Not determined	PI3K/Akt	[158]
	Fetal dermal MSCs	↑Fibroblast migration, proliferation, viability and activity *In vitro*: ↑*collagen I* and *III*, ↑*elastin*, ↑*fibronectin-1*, ↑*αSMA* In vivo: ↑collagen deposition, ECM synthesis	Jagged 1	Notch	[159]
	Adipose tissue MSCs	↑Fibroblast migration, proliferation and invasion	lncRNA H19	Wnt/β-catenin lncRNA H19/miR-19b/SOX9 axis	[160]
	Bone marrow MSCs	↑Fibroblast migration and proliferation, ↑tube formation ↑*c-myc*, ↑*cyclin A1*, ↑*cyclin D2*, ↑*HGF*, ↑*IGF1*, ↑*NGF*, ↑*SDF1*	STAT3	Akt, ERK1/2 and STAT3	[161]
	Adipose tissue MSCs	*In vitro* and *in vivo*: ↑angiogenesis *In vitro*: ↑Tube length and branches number, ↑*Ang1*, ↑*Flk1*, ↓*Vash1* and ↓*TSP1*	miR-125a	suppress DLL4 expression	[162]
	Immortalized adipose MSCs line HATMSC1	↑Proliferation and proangiogenic properties of ECs	EGF, bFGF, IL-8, VEGF, TIMP-1, TIMP-2, miR-210 miR-296, miR-126 miR-378, miR-221, miR-222, miR-92a	VEGFR2	[163]
	Umbilical cord blood	↑Number of new vessels, ↑tube length and branches amount, ↑wound closure, ↑collagen fibers, ↓scar widths, ↓*PTEN* ↓*SPRY1*	miR-21-3p miR-214-5p miR-19b-5p	PI3K/Akt ERK 1/2	[164]
	Urine-derived stem cells	*In vitro*: ↑Fibroblast proliferation and migration In vivo: ↑Pro-angiogenesic effects (number and density of new vessels), ↓scar widths	DMTB1	Not specified	[165]
	Human umbilical cord MSCs	↑Migration, proliferation, and tube formation In vivo: ↑wound closure, ↑angiogenesis	Angiopoietin-1 and 2	Angiopoietin/ TIE	[166]
	Adipose tissue MSCs	↑KCs migration, proliferation, ↑p-AKT, ↑*HIF-1α* In vivo: ↓Wound area	Not determined	AKT/HIF-1α	[167]
	Adipose tissue MSCs	↑KCs proliferation and migration, ↑*MMP-9*, ↑*TIMP2*	miRNA-21	P13K/AKT	[168]
	Adipose tissue MSCs	↑KCs proliferation and migration, ↓apoptosis ↑β-catenin In vivo: ↓wound area	Not determined	Wnt/β-catenin	[169]
	Umbilical cord MSCs	↑Re-epithelization	Wnt4	Wnt/β-catenin	[170]
	Adipose tissue MSCs Bone marrow MSCs	↑Fibroblast, KCs, ECs migration, proliferation, cell viability ↑Tube formation ↑Wound closure	CD73 and various miRNAs	*Both types EVs*: EGFR receptor axis, PI3K/Akt, MAPK, Wnt *Only AdMSCs-EVs*: TGF-β and HIF-1α	[171]
Remodeling	Bone marrow MSCs	*↓TGF-β1*, *↓Smad2*, *↓Smad3*, *↓Smad4*, *↑TGF-**β3*, *↑Smad7*, *↑*α-SMA, ↑VEGF ↑KCs and fibroblast proliferation In vivo: ↑wound healing, ↓wound area, ↑cutaneous appendages	Not determined	TGF-β/Smad	[172]
	Adipose tissue MSCs	ECM remodeling ↑rate of collagen III/collagen I, ↑TGFβ3/TGFβ1, ↑*MMP3* ↑Scarless wound healing	Not determined	ERK/MAPK	[173]
	Adipose tissue MSCs	In vivo: ↑Skin elasticity and barrier integrity, ↑re-epithelization, ↑wound closure, ↑angiogenesis, ↑collagen synthesis ↑*PCNA*, *VEGF*, *filaggrin*, *loricrin*, *AQP3* ↓*TNFα*	Not determined	Not specified	[174]

Abbreviations: Ang-angiopoietin, AQP3-aquaporin-3, Arg1-arginase1, Bcl-2-B-cell lymphoma 2, bFGF-basic fibroblast growth factor, CCL-Chemokine (C-C motif) ligand, DLL4-delta like canonical Notch ligand 4, DMTB1-deleted in malignant brain tumors 1 protein, ECs-endothelial cells, KCs-keratinocytes, ECM-extracellular matrix, EGF-epidermal growth factor, EGRF-epidermal growth factor receptor, ERK-extracellular sig-nal-regulated kinases, EVs-extracellular vesicles, Flk1-receptor protein-tyrosine kinase, HGF-hepatocyte growth factor/scatter factor, HIF-1α-hypoxia-inducible factor 1-alpha, IGF1-insulin-like growth factor 1, IL-interleukin, Jagged1-protein jagged1 precursor, LATS2-large tumor suppressor kinase 2, lncRNA-long non-coding RNA, M1-pro-inflammatory macrophage phenotype, M2-anti-inflammatory mecrophage phenotype, MALAT1-metastasis associated lung adenocarcinoma transcript 1, MAPK-mitogen-activated protein mecrophage phenotype, MALAT1-metastasis associated lung adenocarcinoma transcript 1, MAPK-mitogen-activated protein kinase, Mef2c-myocyte-specific enhancer factor 2C, miR-microRNA, MSCs-mesenchymal stem cells, NF-κβ-nuclear factor kappa-light-chain-enhancer of activated B cells, NGF-nerve growth factor, Notch1-protein neurogenic locus notch homolog protein 1, NOX-NADPH oxidase, Nrf2-nuclear factor erythroid 2-related factor 2, p-AKT-phosphorylated protein kinase B, PCNA-proliferating cell nuclear antigen, MMPs-matrix metalloproteinases, PI3K-phosphatidylinositol 3-kinase, pknox1-PBX/Knotted 1 Homeobox 1, PS—phosphatidylserine, PTEN-phosphatase and tensin homolog, ROS-reactive oxygen species, SDF1-stromal cell-derived factor 1, SMP30-senescence marker protein 30, SOX9-SRY-related high-mobility-group box 9, SPRY1-protein sprouty homolog 1, STAT3-signal transducer and activator of transcription 3, TF—tissue factor, TGF-β-transforming growth factor beta, TIMP1-metallopeptidase inhibitor 1 precursor, TLR4-toll-like receptor 4, TNF-tumor necrosis factor, TSP1-thrombospondin 1, Vash1-tubulin carboxypeptidase 1, VEGFR2-vascular endothelial growth factor receptor 2, VEGF-vascular endothelial growth factor, α-SMA-α smooth muscle actin. Italic font means changes in gene expression.

**Table A3:** Plant-derived EV role in wound healing.

Phase	EVs Source	Effects	EVs Molecules Involved	Signalling Pathway	Reference
Inflammation	*Citrus limon L.*	Anti-oxidative effect on mesenchymal stromal cells	Citrate Vitamin C Short miRNAs with unknown function	Not specified	[183]
	Strawberry juice *Fragaria x ananassa*	↓ROS production	Vitamin C Small RNAs (17–30 nt) miR166g	Not specified	[184]
	Blueberry	↓Oxidative stress ↓ROS levels ↑Antioxidant genes expression ↑Bcl2 ↑HO-1 ↓Bax ↓FAS ↓ACC1	Not determined	Nrf2	[185]
	Blueberry	↓TNFα induced ROS generation ↑cell viability	miR-156e, miR-162, and miR-319d	Involved in 340 canonical pathways, 121 KEGG pathways, and 121 GO Biological processes	[186]
	Grape	↓DSS induced colitis	Not determined	Wnt/β-catenin pathway	[187]
	*Curcuma Longa*	↓colitis ↑promote intestinal wound repair ↑HO-1 ↓IL-6 ↓IL-1β ↓TNF-α	Not determined	Nf-κB pathway	[188]
	Ginger	↑HO-1 ↑IL-10 ↑IL-6 ↑nuclear factor like (erythroid-derived 2)	Not determined	Wnt/TCF4	[189]
	Grapefruit	↑nuclear factor like (erythroid-derived 2)	Not determined	Wnt/TCF4	[189]
	Carrot	↑nuclear factor like (erythroid-derived 2)	Not determined	Wnt/TCF4	[189]
	Ginger	↓lung inflammation ↓Nsp12	aly-miR396a-5p and rlcv-miR-rL1-28-3p	NK-κB	[190]
Proliferation	Grapefruit	↑HaCaT cells’ viability ↑cell migration ↓intracellular ROS production ↑HUVECs tube formation capabilities	Not determined	Not specified	[192]
	Wheat (*Triticum aestivum*)	↑endothelial, epithelial and dermal fibroblast cells’ viability and migration ↑vascularization and angiogenesis	Not determined	Not specified	[177]
	Ginger	↑intestinal wound healing ↓TNF-α, IL-6 and IL-1β ↑IL-10 and IL-22	phosphatidic acid, digalactosyldiacylglycerol, monogalactosyldiacyglycerol, actin and proteolysis enzymes, aquaporin and chloride channels, ~125 miRNAs, 6-gingerol, 6-shogaol	Not specified	[193]

Abbreviations: ACC1-acetyl-Coa carboxylase 1, Bax-BCL2-associated protein, Bcl-2-B-cell lymphoma 2, EVs-extracellular vesicles, FAS-Fas cell surface death receptor, HaCaT-human epidermal keratinocyte cell line, HO-1-heme oxygenase-1, HU-VECs-human umbilical vein endothelial cells, IL-interleukin, KEGG-Kyoto Encyclopedia of Genes and Genomes, miR-microRNA, NF-κβ-nuclear factor kappa-light-chain-enhancer of activated B cells, Nrf2-nuclear factor erythroid 2-related factor 2, Nsp12-SARS-CoV-2 nonstructural protein 12, ROS-reactive oxygen species, TNF-tumour necrosis factor.
